# Intratumoral and peritumoral PET/CT-based radiomics for non-invasively and dynamically predicting immunotherapy response in NSCLC

**DOI:** 10.1038/s41416-025-02948-z

**Published:** 2025-02-10

**Authors:** Xianwen Lin, Zhiwei Liu, Kun Zhou, Yuedan Li, Genjie Huang, Hao Zhang, Tingting Shu, Zhenhua Huang, Yuanyuan Wang, Wei Zeng, Yulin Liao, Jianping Bin, Min Shi, Wangjun Liao, Wenlan Zhou, Na Huang

**Affiliations:** 1 Department of Oncology, Nanfang Hospital, Southern Medical University, Guangzhou, China; 2 Cancer Center, the Sixth Affiliated Hospital, School of Medicine, South China University of Technology, Foshan, China; 3 Foshan Key Laboratory of Translational Medicine in Oncology, the Sixth Affiliated Hospital, School of Medicine, South China University of Technology, Foshan, China; 4 Nanfang PET Center, Nanfang Hospital, Southern Medical University, Guangzhou, China; 5 Department of Cardiology, Nanfang Hospital, Southern Medical University, Guangzhou, China

**Keywords:** Tumour biomarkers, Cancer imaging, Machine learning, Predictive medicine

## Abstract

**Background:**

We aimed to develop a machine learning model based on intratumoral and peritumoral ^18^F-FDG PET/CT radiomics to non-invasively and dynamically predict the response to immunotherapy in non-small cell lung cancer (NSCLC).

**Methods:**

This retrospective study included 296 NSCLC patients, including a training cohort (N = 183), a testing cohort (N = 78), and a TCIA radiogenomic cohort (N = 35). The extreme gradient boosting algorithm was employed to develop the radiomic models.

**Results:**

The COMB-Radscore, which was developed by combining radiomic features from PET, CT, and PET/CT images, had the most satisfactory predictive performance with AUC (ROC) 0.894 and 0.819 in the training and testing cohorts, respectively. Survival analysis has demonstrated that COMB-Radscore is an independent prognostic factor for progression-free survival and overall survival. Moreover, COMB-Radscore demonstrates excellent dynamic predictive performance, with an AUC (ROC) of 0.857, enabling the earlier detection of potential disease progression in patients compared to radiological evaluation solely relying on tumor size. Further radiogenomic analysis showed that the COMB-Radscore was associated with infiltration abundance and functional status of CD8 + T cells.

**Conclusions:**

The radiomic model holds promise as a precise, personalized, and dynamic decision support tool for the treatment of NSCLC patients.

## Background

Immunotherapies that target programmed death 1/programmed death ligand 1 (PD1/PD-L1) have become the standard treatment for locally advanced or metastatic non-small cell lung cancer (NSCLC) [1, 2]. However, despite breakthroughs in immunotherapy that have improved prognosis in advanced NSCLC patients, only a subset of these patients experience long-term clinical benefits [3, 4]. Therefore, there is an urgent need to identify an effective biomarker for predicting immunotherapy efficacy.

PD-L1 is currently the most widely utilized biomarker for predicting the effectiveness of anti-PD1/PD-L1 immunotherapy [5]. However, PD-L1 is not a perfect predictor of the efficacy of this treatment [6]. Clinical trials have demonstrated that some patients who test negative for PD-L1 can still derive benefits from anti-PD1/PD-L1 therapy, which raises concerns about the predictive value of PD-L1 [7, 8]. Furthermore, the detection of PD-L1 expression is influenced by spatial and temporal heterogeneity, suggesting that a single biopsy may not accurately represent the overall and dynamic expression of PD-L1 throughout the entire tumor [9]. Last but not least, detecting PD-L1 requires invasive histological biopsy sampling, which may be hindered by patient tolerance and the quantity and quality of the specimens obtained. Therefore, it is imperative to further develop non-invasive, stable, and accurate biomarkers for predicting the effectiveness of immunotherapy.

Previous studies have demonstrated the potential of fluoro-18-fluorodeoxyglucose positron emission tomography/computed tomography (^18^F-FDG PET/CT) radiomics in various aspects of NSCLC, including preoperative lymph node staging, prediction of epidermal growth factor receptor mutation status, and evaluating the tumor immune microenvironment [10–12].

However, previous radiomics studies have primarily focused on the tumor itself, overlooking the potential information that may exist in the peri-tumoral regions. An increasing number of studies have confirmed that the imaging characteristics of the regions surrounding tumors may provide valuable information relevant to tumor diagnosis, treatment efficacy, and prognosis, which are essential for predictive modeling [13, 14]. Further comprehensive and in-depth research on tumor and peritumoral radiomics will help us gain a better understanding of the complex heterogeneity of tumors.

Moreover, the majority of research conducted previously on radiomics has focused on baseline or single-time-point medical images, neglecting the analysis of dynamic images at various time points during treatment. Predictive models developed based on radiomics often lack an exploration of their dynamic prediction capability, making it difficult for them to cope with complex and variable clinical scenarios. Therefore, the development of a radiomic model with dynamic prediction capability can enhance its practical application in complex and variable clinical scenarios, providing more precise and targeted treatment and follow-up strategies for patients.

In this study, we developed, validated, and applied a machine learning model based on intratumoral and peritumoral ^18^F-FDG PET/CT radiomics to non-invasively and dynamically predict prognosis and response to immunotherapy in NSCLC patients.

## Methods

### Study design and participant cohorts

**Fig. 1:**
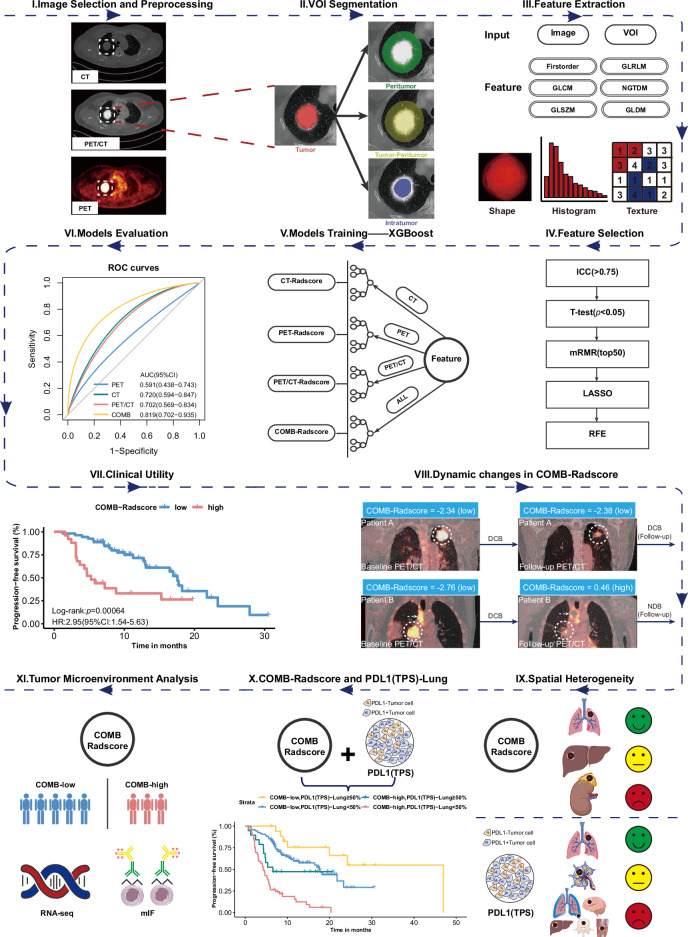
Overall study design. VOI volume of interest, GLCM gray-level co-occurrence matrix, GLDM gray-level dependence matrix, GLRLM gray-level run-length matrix, GLSZM gray-level size-zone matrix, NGTDM neighboring gray-tone difference matrix, ICC intraclass/interclass correlation coefficient, mRMR minimum redundancy maximum relevance, LASSO least absolute shrinkage and selection operator, RFE recursive feature elimination, XGBoost extreme gradient boosting, COMB-Radscore combined Radscore, ROC curves receiver operating characteristic curves, AUC area under the curve, CI confidence interval, HR hazard ratio, DCB durable clinical benefit, NDB no durable clinical benefit, PD-L1 (TPS) programmed cell death ligand 1 (tumor proportion score), RNASeq RNA sequencing, mIF multiplex immunofluorescence.

Figure 1 outlines the overall study design. In this study, we retrieved data from the database of Nanfang Hospital, Southern Medical University, covering the period from January 2018 to January 2023. A total of 261 patients diagnosed with NSCLC and received to first-line anti-PD1/PD-L1 immunotherapy were identified. The specific inclusion and exclusion criteria are detailed in Fig. S1. A simple random sampling strategy was implemented, dividing patients into a training cohort and a testing cohort in a 7:3 ratio. The reproducibility of the randomization process was ensured by utilizing a predefined random seed. Subsequently, radiomics analysis was conducted on both cohorts.

To further explore the biological basis of the radiomics model, an internal radiogenomics cohort was established by selecting 41 patients with available RNA sequencing data from both the training and testing cohorts. To form an independent external radiogenomic cohort, we recruited 35 eligible NSCLC patients from the TCIA database, which stores the TCGA-LUAD [15] (10.7937/K9/TCIA.2016.JGNIHEP5) and TCGA-LUSC [16] (10.7937/K9/TCIA.2016.TYGKKFMQ) datasets, and performed radiogenomic analysis on the internal and external radiogenomic cohorts. To further validate the conclusions of the radiogenomics analysis, a multiplex immunofluorescence cohort was formed by selecting 31 patients with available matched pathological tissue sections from both the training and testing cohorts. The detailed flowchart of patient selection can be found in Fig. S1.

This retrospective study was approved by the ethics committee of Nanfang Hospital of Southern Medical University (NFEC-2019-265). Informed consent was obtained from the patients for the use of pathological specimens, while the necessity for patient informed consent for the use of clinical data, laboratory test results, and ^18^F-FDG PET/CT was waived.

### Data collection

We evaluated and collected best overall response, progression-free survival (PFS), and overall survival (OS) in the follow-up phase of treatment. The definitions of complete response (CR), partial response (PR), stable disease (SD), and progressive disease (PD) were based on the RECIST V.1.1 criteria. The primary endpoint of this study was the clinical benefit of immunotherapy, defined as durable clinical benefit (DCB) lasting ≥ 6 months, which included CR, PR, or SD ≥ 6 months, or no durable clinical benefit (NDB) with PD or SD lasting <6 months. The remaining collected clinicopathological characteristics and laboratory test data are described in detail in the supplementary methods.

### Radiomic workflow

In this study, we developed a radiomic workflow for ^18^F FDG PET/CT images. First, we extracted DICOM-format data from the pre-treatment CT scans and the corresponding PET scans for each patient. The CT scans provide information on anatomical morphology, while the PET scans offer insights into the functional metabolism of the lesions. Subsequently, a strategy for image fusion was developed to integrate the respective strengths of PET and CT imaging while addressing the limitations inherent in single-modality images. Through this approach, PET and CT images were combined to produce PET/CT images that encompass both detailed anatomical information and functional metabolic data.

In the segmentation phase, a multi-volume-of-interest (VOI) segmentation strategy was employed to comprehensively characterize the tumor. Specifically, four distinct VOIs were defined: tumoral, peritumoral, intratumoral, and tumor–peritumoral volumes. Through this workflow, a total of 12 VOIs were obtained, with four VOIs corresponding to each image type. Subsequently, radiomic features were extracted from PET, CT, and PET/CT images using PyRadiomics in Python. Each VOI within a single image type generated 2060 radiomic features, resulting in a total of 8240 features for each imaging modality.

To mitigate the curse of dimensionality, prevent model overfitting, and enhance robustness, a comprehensive feature filtering process was implemented. This process included the use of intra- and inter-class correlation coefficients (ICCs), Student’s t-tests, minimum redundancy maximum relevance (mRMR), least absolute shrinkage and selection operator (LASSO), and recursive feature elimination (RFE).

A detailed description of the methods used for image acquisition, image fusion, image segmentation, image preprocessing, feature extraction, and feature selection in the workflow of radiomics is provided in Supplementary methods.

The PET, CT, and PET/CT images went through the above workflow, and the following four prediction models were established using an extreme gradient boosting (XGBoost) algorithm:

I. PET-Radscore: Includes features extracted from PET images.
II. CT-Radscore: Includes features extracted from CT images.
III. PET/CT-Radscore: Includes features extracted from PET/CT images.
IV. Combined (COMB)-Radscore: Includes features extracted from PET, CT, and PET/CT images.

After the model training, a 10-fold cross-validation grid-search method was used to fine-tune the model parameters. The features and parameters of each model are described in Table S1. Following their development, a comprehensive performance evaluation of each model was conducted, as detailed in the supplementary methods.

### Statistical analysis

All statistical and machine learning analyses were performed using R version 4.3.0 (The R Project for Statistical Computing, http://www.r-project.org/). The statistical tests were two-sided, with a p-value significance threshold of 0.05 adopted throughout. Student’s *t*-test was used to test for differences in continuous variables, and the χ2 test or Fisher’s exact test was used to test for differences in categorical variables. The cutoff value was established using the maximum Youden index (i.e., Specificity + Sensitivity − 1) in the training cohort. Subsequently, this cutoff value from the training cohort was applied consistently to the other cohorts. When evaluating performance, the models maintained a consistent cutoff value across all cohorts. Patients were classified into two categories based on the calculated cutoff. Survival analyses were performed using a Cox proportional hazard model, Kaplan-Meier survival estimates, and the log-rank test. The Pearson correlation coefficient was used to measure the correlations between two variables.

## Results

### Demographic and clinicopathological characteristics

The demographic and clinicopathological characteristics of the training cohort and the testing cohort are presented in Table S2. The training and testing cohorts consisted of 183 and 78 patients, respectively. Among them, DCB was achieved by 184 patients (70.5%), while the remaining 77 patients (29.5%) did not achieve DCB. No statistically significant differences were observed between the two cohorts concerning the baseline characteristics in this study. Among all enrolled patients, those who achieved DCB demonstrated several distinguishing characteristics when compared to those who achieved NDB. These factors included elevated levels of body mass index, PD-L1 tumor proportion score (TPS), and albumin, as well as decreased levels of serum cytokeratin 19 fragment antigen 211, neuron-specific enolase, squamous cell carcinoma antigen, platelets, and C-reactive protein (all p < 0.05).

### Performance evaluation of the prediction models

**Fig. 2:**
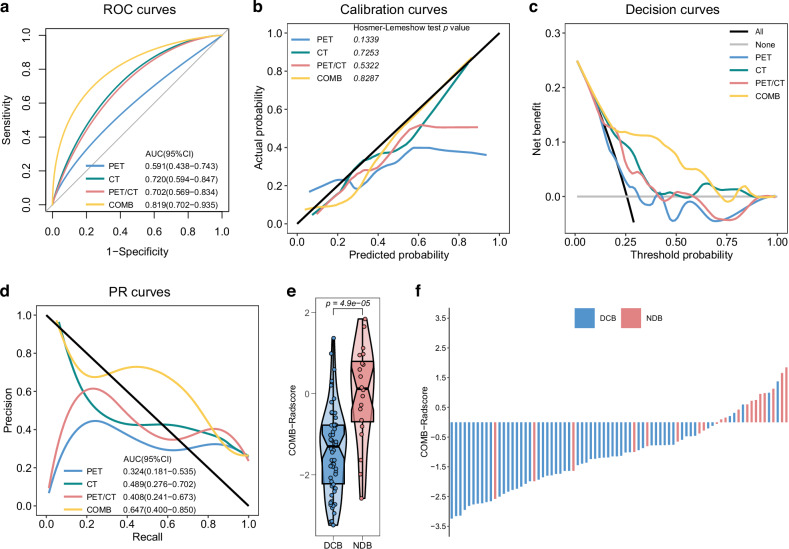
Performance evaluation of prediction models. ROC curves (**a**), calibration curves (**b**), decision curves (**c**) and PR curves (**d**) of the four radiomic models in the testing cohort. **e** Difference in COMB-Radscore between DCB and NDB groups in the testing cohort. **f** Response (DCB/NDB) and COMB-Radscore for each patient in the testing cohort. ROC curves receiver operating characteristic curves, PR curves precision-recall curves, AUC area under the curve, CI confidence interval, DCB durable clinical benefit, NDB no durable clinical benefit.

We followed a predetermined radiomic workflow and developed the PET-Radscore, CT-Radscore, PET/CT-Radscore, and COMB-Radscore models. Subsequently, the radiomic model with the highest predictive ability was determined through a comprehensive evaluation of model performance. The receiver operating characteristic (ROC) curve shows that the COMB-Radscore model had the highest the area under the curve (AUC) value among all evaluated models. The AUC (ROC) values for the PET-Radscore, CT-Radscore, PET/CT-Radscore, and COMB-Radscore models in the training cohort were 0.846 (p-value < 0.0001), 0.856 (p-value < 0.0001), 0.768 (p-value < 0.0001), and 0.894 (p-value < 0.0001), respectively (Fig. S2a). In the testing cohort, the AUC values were 0.591 (p-value = 0.2295), 0.720 (p-value = 0.0035), 0.702 (p-value = 0.0074), and 0.819 (p-value < 0.0001), respectively (Fig. 2a).

The calibration curve showed a good fit of the COMB-Radscore, with no significant differences observed in either the training or testing cohort according to the Hosmer–Lemeshow test (Figs. S2b and 2b). Decision curve analysis revealed that all models achieved net clinical benefit against a treat-all-or-none plan, and the COMB-Radscore exhibited the highest net benefit across most threshold probability ranges (Figs. S2c and 2c). The precision-recall (PR) curve demonstrated that the COMB-Radscore achieved the highest AUC (PR) in both the training and testing cohorts, with values of 0.819 (p-value < 0.0001) and 0.647 (p-value = 0.0209), respectively (Figs. S2d and 2d).

In the testing cohort, the COMB-Radscore demonstrated superior prediction accuracy compared to the PET-Radscore (NRI = 0.34, *p*_NRI_ = 0.035, IDI = 0.21, *p*_IDI_ = 0.007), CT-Radscore (NRI = 0.29, *p*_NRI_ = 0.038, IDI = 0.14, *p*_IDI_ = 0.012), and PET/CT-Radscore (NRI = 0.25, *p*_NRI_ = 0.111, IDI = 0.17, *p*_IDI_ = 0.006) based on the net reclassification improvement (NRI) and integrated discrimination improvement (IDI) analyses (Table S3). Furthermore, the evaluation metrics, including positive predictive value (PPV), negative predictive value (NPV), sensitivity, specificity, accuracy (ACC), recall, F1 score, Matthews correlation coefficient (MCC), and Kappa, also indicated that the COMB-Radscore exhibited optimal predictive performance (Table S4).

Upon further analysis of the relationship between the COMB-Radscore and treatment response, significant statistical differences in the COMB-Radscore were observed among the different treatment responses. The groups with better treatment responses (DCB, CR/PR, CR/PR/SD) exhibited lower COMB-Radscore (Figs. S2e, 2e, and S3). The COMB-Radscore and therapeutic response (DCB/NDB) of each patient in the training and testing cohorts are illustrated in Figs. S2f and 2f, respectively.

Furthermore, the predictive performance of COMB-Radscore was compared to that of 10 serum inflammatory markers, and it was found that COMB-Radscore outperformed all other markers (Fig. S4a, b). Correlation analysis revealed no significant association between COMB-Radscore and 10 serum inflammatory markers (Fig. S4c).

Collectively, these results indicated superior performance of the COMB-Radscore model in comparison to the other radiomic models or serum inflammatory markers.

### Clinical utility of the COMB-Radscore

**Fig. 3:**
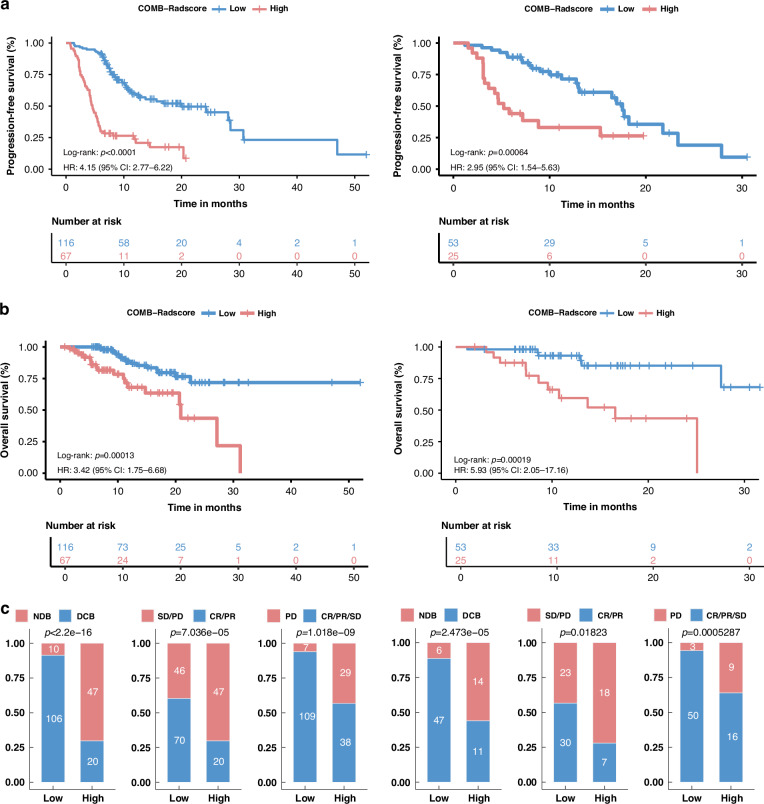
Clinical utility of the COMB-Radscore. Kaplan-Meier analysis of PFS (**a**) and OS (**b**) for the low and high COMB-Radscore groups in the training (left) and testing (right) cohorts. **c** Proportional composition of different patient responses between the low and high COMB-Radscore groups in the training (left) and testing (right) cohorts. HR hazard ratio, CI confidence interval, PFS progression-free survival, OS overall survival, CR complete response, PR partial response, SD stable disease, PD progressive disease. The definitions of CR, PR, SD, and PD were based on the RECIST V.1.1 criteria.

To further explore the clinical applicability of the COMB-Radscore, we conducted an analysis to compare the low and high COMB-Radscore groups in terms of PFS, OS, and response to immunotherapy. The Kaplan-Meier survival curves revealed significant differences in PFS (training cohort log-rank test p-value < 0.0001; testing cohort log-rank test p-value = 0.00064) and OS (training cohort log-rank test p-value = 0.00013; testing cohort log-rank test p-value = 0.00019) between the low and high COMB-Radscore groups (Fig. 3a, b). Patients in the low COMB-Radscore group exhibited prolonged PFS and OS. In addition, a higher proportion of patients with DCB, CR/PR, or CR/PR/SD were observed within the low COMB-Radscore group compared to the high COMB-Radscore group (Fig. 3c).

Univariate Cox regression analysis was performed on all baseline clinicopathological variables to predict PFS and OS, followed by multivariate Cox regression analysis, which included variables with a p-value less than 0.05 to control for potential confounders. The COMB-Radscore remained a powerful and independent prognostic factor for predicting both PFS and OS (Tables S5–8).

In the PFS analysis, additional subgroup analyses were conducted based on various clinical and pathological variables. When stratified by factors such as gender, age, smoking status, histological type, T stage, N stage, M stage, overall stage, number of metastases, treatment strategy, and irAE, the COMB-Radscore remained a statistically significant prognostic classifier in most subgroups (Tables S9, 10).

Overall, our findings further confirm significant differences in patient prognosis when stratified by the COMB-Radscore.

### Dynamic predictive ability of the COMB-Radscore

The performance of the COMB-Radscore in dynamically predicting subsequent treatment efficacy was validated using the follow-up ^18^F-FDG PET/CT scans of patients. In this part of the study, 25 patients from the training and testing cohorts were included, all of whom underwent follow-up ^18^F-FDG PET/CT scans 6 to 12 months post-treatment. Based on disease progression within 6 months after follow-up ^18^F-FDG PET/CT, we categorized the patients into two groups: the NDB (Follow-up) group consisting of 11 patients with disease progression and the DCB (Follow-up) group consisting of 14 patients without disease progression. The detailed procedures for validating the model’s dynamic predictive capabilities are provided in the supplementary methods.

**Fig. 4:**
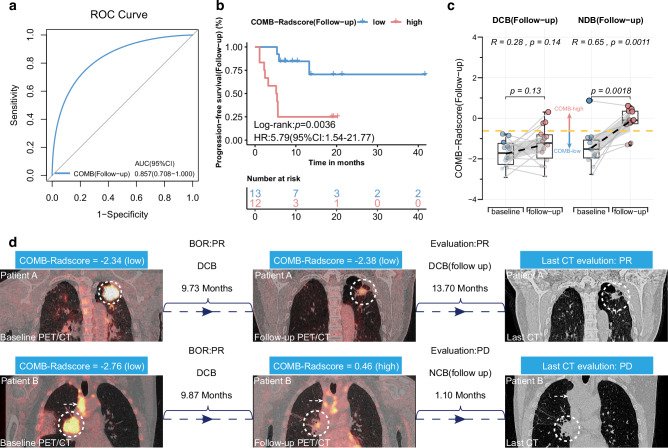
Dynamic predictive ability of and changes in COMB-Radscore. **a** ROC curve of COMB-Radscore (Follow-up) in the follow-up cohort. **b** Kaplan-Meier analysis of PFS (Follow-up) for low and high COMB-Radscore (Follow-up) groups in the follow-up cohort. **c** Changes in COMB-Radscore of patients in the DCB (Follow-up) group and NDB (Follow-up) group. **d** Changes in COMB-Radscore of two representative patients during treatment. ROC curve receiver operating characteristic curve, AUC area under the curve, CI confidence interval, HR hazard ratio, DCB durable clinical benefit, NDB no durable clinical benefit, PFS progression-free survival, BOR best overall response, PR partial response, PD progressive disease.

The ROC and PR curves demonstrated a favorable predictive ability of the COMB-Radscore (Follow-up), yielding AUC values of 0.857 (p-value = 0.0026) for the ROC and 0.836 (p-value = 0.0046) for the PR curves, respectively (Figs. 4a and S5c). The calibration and decision curves also demonstrated good calibration and clinical applicability of the COMB-Radscore (Follow-up) (Fig. S5a, b). Likewise, the COMB-Radscore (Follow-up) exhibited strong performance in other model evaluation metrics (Table S11). Significant differences were observed among different subsequent treatment outcomes with respect to the COMB-Radscore (Follow-up), with lower levels observed in the DCB (Follow-up) group (Fig. S5d).

We analyzed the clinical utility of the COMB-Radscore (Follow-up). PFS (Follow-up) was defined as the duration from the initiation of ^18^F-FDG PET/CT follow-up scans to disease progression. Survival analysis showed a significant difference in PFS (follow-up) between the low and high COMB-Radscore (Follow-up) groups, with the low COMB-Radscore (Follow-up) group demonstrating prolonged PFS (Follow-up) compared to its counterparts (log-rank test p-value = 0.0036) (Fig. 4b). There was a higher proportion of patients achieving DCB (Follow-up) in the low COMB-Radscore (Follow-up) group compared to the other groups (Fig. S5e).

Subsequently, we conducted further analysis of the dynamic changes in COMB-Radscore. In the NDB (Follow-up) group, there was a significant increase in COMB-Radscore based on follow-up ^18^F-FDG PET/CT images compared to the baseline. However, no significant changes were observed in the DCB (Follow-up) group (Fig. 4c).

Figure 4d illustrates the radiological responses and the corresponding changes in COMB-Radscore for two representative patients from the previously mentioned retrospective cohort.

Patient A underwent a baseline ^18^F-FDG PET/CT scan prior to initiating first-line treatment, which yielded a baseline COMB-Radscore of −2.34 (COMB-low). The best overall response (BOR) to immunotherapy was a PR, classified as DCB, indicating that the baseline COMB-Radscore effectively predicted Patient A’s response to immunotherapy. After 9.73 months of treatment, a follow-up ^18^F-FDG PET/CT scan revealed a radiological PR, with the COMB-Radscore (follow-up) remaining stable at −2.38, showing no significant change from the baseline. This stability suggests that Patient A had a low risk of subsequent tumor progression and could continue to benefit from immunotherapy. As anticipated, Patient A continued to receive immunotherapy for an additional 13.70 months, with regular radiological evaluations confirming PR until they were lost to follow-up.

Similarly, Patient B also underwent a baseline ^18^F-FDG PET/CT scan prior to initiating first-line therapy, resulting in a COMB-Radscore of −2.76 (COMB-low). The BOR to immunotherapy was a PR, classified as DCB, indicating that the baseline COMB-Radscore also successfully predicted Patient B’s response to immunotherapy. After 9.87 months of treatment, Patient B underwent a follow-up ^18^F-FDG PET/CT scan. At this time, the follow-up COMB-Radscore had significantly increased to 0.46 (COMB-high), indicating a high risk of subsequent tumor progression, despite the radiological evaluation still showing a PR at that time. As anticipated, disease progression was detected during the follow-up CT scan after Patient B received two additional cycles of immunotherapy.

These findings suggest that, compared to relying solely on tumor size for radiological evaluation, the COMB-Radscore has the potential to facilitate the early detection of disease progression in patients.

### Complementarity of COMB-Radscore and TPS-Lung

In this study, we further explored the spatial heterogeneity of the predictive capabilities of TPS and COMB-Radscore. The results showed that the TPS derived from biopsy specimens of primary lung tumors, designated as TPS-Lung, demonstrated superior predictive performance for immunotherapy efficacy compared to TPS derived from other regions. Likewise, the COMB-Radscore derived from primary lung tumors demonstrated superior predictive performance for the efficacy of immunotherapy compared to the COMB-Radscore derived from metastases at other locations. Detailed results regarding spatial heterogeneity are presented in the supplementary materials (Supplementary Results 1, Figs. S6–S8, Tables S12, 13).

In this study, both the TPS-lung and COMB-Radscore showed strong predictive capabilities for the efficacy of immunotherapy. Consequently, we further explored the correlation and potential complementarity between them. The correlation analysis revealed no significant association between COMB-Radscore and TPS-Lung, and there was no statistically significant difference in the distribution of TPS-Lung between the low and high COMB-Radscore groups (Fig. S9).

**Fig. 5:**
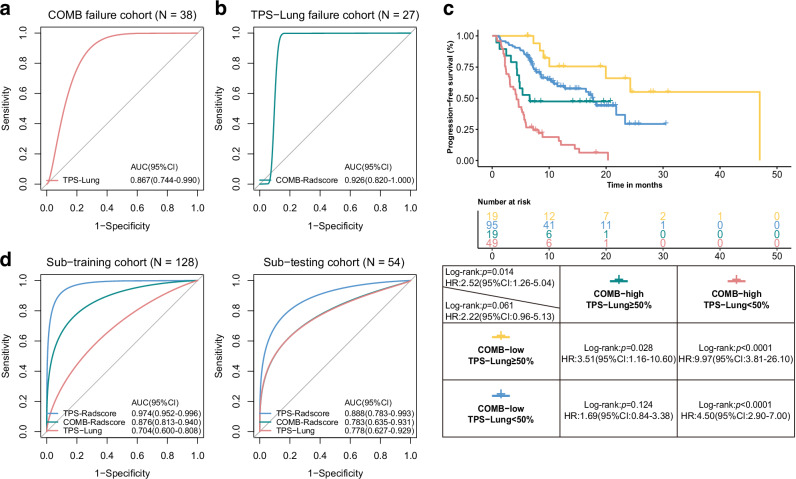
Complementarity between COMB-Radscore and TPS-Lung. **a** ROC curve of TPS-Lung in the COMB-Radscore prediction failure cohort. **b** ROC curve of COMB-Radscore in the TPS-Lung prediction failure cohort. **c** Kaplan-Meier analysis of PFS of four groups of patients stratified by COMB-Radscore and TPS-Lung. **d** ROC curves of the TPS-Radscore, COMB-Radscore, and TPS-Lung in the sub-training (left) and sub-testing (right) cohorts. ROC curves, receiver operating characteristic curves, AUC, area under the curve, CI confidence interval, HR hazard ratio, PFS progression-free survival, TPS tumor proportion score.

Subsequently, two cohorts were established from the training and the testing cohorts. The first cohort, referred to as the COMB-Radscore prediction failure cohort, consisted of NDB patients with a low COMB-Radscore and DCB patients with a high COMB-Radscore. In this particular cohort, the AUC (ROC) for TPS-Lung was found to be 0.867 (p-value = 0.0002) (Fig. 5a). The second cohort, known as the TPS-Lung prediction failure cohort, included NDB patients with a TPS-Lung ≥ 50% and DCB patients with a TPS-Lung <1%. Within this specific cohort, the AUC (ROC) for the COMB-Radscore was calculated to be 0.926 (p-value = 0.0004) (Fig. 5b). Furthermore, when combining both COMB-Radscore and TPS-lung assessments, a more refined stratification of patients was achieved (Figs. 5c and S10). Specifically, individuals classified as COMB-low (low COMB-Radscore) + TPS-Lung ≥50% demonstrated significant benefits from immunotherapy in terms of a higher proportion of DCB patients and longer PFS and OS. Conversely, those categorized as COMB-high (high COMB-Radscore) + TPS-Lung <50% exhibited the opposite outcomes.

In the NSCLC population with a TPS < 50%, there is currently no recognized biomarker to distinguish the patient population that would benefit from immunotherapy monotherapy or combination therapy. Therefore, we further evaluated the potential application value of COMB-Radscore in this clinical scenario. Survival analysis revealed that in the low COMB-Radscore group, combination therapy did not offer a long-term PFS benefit over monotherapy. In contrast, patients in the high COMB-Radscore group who received combination therapy experienced a significant improvement in PFS compared to those on monotherapy. Detailed results are presented in the supplementary materials (Supplementary Results 2, Fig. S11). Subsequently, to develop an integrated model, we created a new sub-training and sub-testing cohort by selecting 128 and 54 patients with TPS-Lung information from the training and testing cohorts, respectively. In the sub-training cohort, an integrated model named TPS-Radscore was developed using the XGBoost algorithm by combining COMB-Radscore and TPS-Lung. The predictive ability of TPS-Radscore was significantly improved compared to COMB-Radscore or TPS-Lung alone. In the sub-training and sub-testing cohorts, the AUC (ROC) values for identifying DCB patients were 0.974 (p-value < 0.0001) and 0.888 (p-value < 0.0001), respectively (Fig. 5d). In the sub-testing cohort, NRI and IDI analyses demonstrated that the TPS-Radscore exhibited higher prediction accuracy than the COMB-Radscore (Table S14). Furthermore, other model evaluation metrics, were superior to those of COMB-Radscore as well (Table S15).

### Biological basis of the COMB-Radscore

Radiogenomic analysis was conducted to explore the underlying biological basis of the COMB-Radscore. First, gene set enrichment analysis [17] (www.gsea-msigdb.org/gsea/index.jsp) was performed to identify potential molecular pathways associated with the COMB-Radscore. Significant enrichment in several immune-related molecular pathways was observed in the COMB-low group. (Fig. S12a). The TCIA cohort also exhibited similar findings (Fig. S12b).

**Fig. 6:**
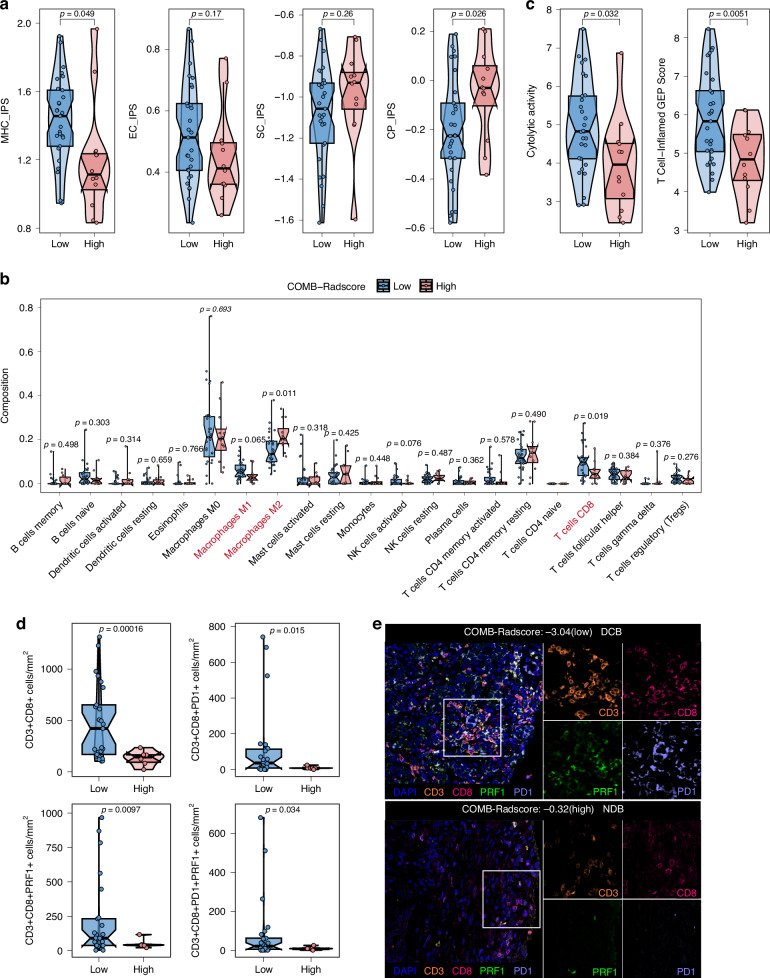
Differences in the tumor immune microenvironment between low and high COMB-Radscore groups. Difference in four immune phenotype scores (**a**), abundances of 22 immune cells (**b**), cytolytic activity and T-cell–inflamed GEP score (**c**) between the low and high COMB-Radscore groups in the Internal radiogenomics cohort. **d** Difference in CD3 + CD8 + T cells, CD3 + CD8 + PRF1 + T cells, CD3 + CD8 + PD1 + T cells, and CD3 + CD8 + PD1 + PRF1 + T cells between the low and high COMB-Radscore groups in the Multiple immunofluorescence cohort. **e** Micrographs of the multiplex immunofluorescence for two representative patients. MHC major histocompatibility complex, EC effector cells, SC suppressor cells, CP checkpoints, IPS immunophenoscore, GEP gene expression profile, DAPI 4′,6-diamidino-2-phenylindole, CD3 cluster of differentiation 3, CD8 cluster of differentiation 8, PRF1 perforin-1, PD1 programmed cell death protein 1.

Subsequently, we analyzed the differences in the immune microenvironment between low and high COMB-Radscore patients using IOBR [18], an immunology tool previously developed by our research group. We calculated the four different immune phenotypes (MHC molecules, effector cells, suppressor cells, and checkpoints) using IPS [19]. The results showed that the COMB-low group demonstrated higher scores for MHC molecules (p = 0.049) and lower scores for checkpoints (p = 0.026) compared to the COMB-high group (Fig. 6a). Additionally, we evaluated the infiltration abundance of immune cells in patients using Cibersort [20] and further analyzed the T cell functional status of the two groups of patients using gene markers for cytolytic activity(CYT) [21] and the T-cell-inflamed gene-expression profile (GEP) [22]. The results revealed that the COMB-low group exhibited elevated levels of CD8 + T cells (p = 0.019) and M1 macrophages (p = 0.065), while displaying decreased levels of M2 macrophages (p = 0.011) (Fig. 6b). Furthermore, the COMB-low group demonstrated higher CYT (p = 0.032) and an enhanced T-cell–inflamed GEP score (p = 0.0051) compared to the COMB-high group (Fig. 6c). Similar results were also observed in the TCIA cohort (Fig. S13).

The composition of CYT genes was analyzed, revealing a significantly higher expression level of the PRF1 (p = 0.031) gene in the COMB-low group than in the COMB-high group (Fig. S14a). However, no significant difference was observed in the expression level of the GZMA (p = 0.26) gene. Similarly, we examined the expression levels of the immune checkpoint PDCD1 (p = 7.5e-05) and found a significant upregulation in its expression within the COMB-low group. Furthermore, correlation analysis demonstrated a significant negative association between PRF1 and PDCD1 with respect to the COMB-Radscore (Fig. S14c). Similar results were also observed in the TCIA cohort (Fig. S14b, d).

To further validate the disparities in CD8 + T cell quantity and function within the tumor immune microenvironment between the two patient groups, we conducted multiplex immunofluorescence staining on pathological tissue sections from 31 patients in the training and testing cohorts. The results showed a higher density of CD3 + CD8 + T cells, CD3 + CD8 + PRF1 + T cells, CD3 + CD8 + PD1 + T cells, and CD3 + CD8 + PD1 + PRF1 + T cells (all p < 0.05) in the COMB-low group compared to the COMB-high group (Fig. 6d). These findings suggest that patients with a low COMB-Radscore exhibit an immune-inflamed tumor microenvironment and are more likely to benefit from immunotherapy.

## Discussion

Previous studies have demonstrated the effectiveness of radiomics as a non-invasive method for predicting the response of NSCLC patients to anti-PD1/PDL-1 therapy. Wu et al. extracted radiomic features from thin-slice chest CT images of NSCLC patients before immunotherapy and used LASSO and stepwise logistic regression to establish a combined radiomic signature for predicting immunotherapy response [23]. The combined radiomic signature identified DCB patients with an AUC (ROC) of 0.82 and 0.75 in the training and validation cohorts, respectively.

Radiomics offers unique insights into tumor biology and treatment responses. However, previous radiomics studies often focused on single images or single VOI, difficult to comprehensively characterize the heterogeneity of tumors and their tumor microenvironment. Moreover, it is crucial to meticulously and transparently report all parameters used in the radiomics workflow, including pre-processing and PyRadiomics steps, to ensure standardized methodologies [24]. This will guarantee the consistency of feature extraction, extremely improve model robustness and reproducibility, facilitate comparisons across studies. Therefore, in this study, we developed an ^18^F-FDG PET/CT radiomics machine learning model using multi-image fusion and multi-VOI segmentation strategies to predict immunotherapy response, and reported the parameters and methodologies used in the radiomics workflow minutely and clearly, including scanning equipment, pre-processing, standardization, fusion, segmentation, feature extraction, feature filtering, and modeling. Results showed that the COMB-Radscore, which was developed by integrating radiomic features from PET, CT, and PET/CT images, had the most satisfactory predictive performance.

In machine learning, imbalanced data in classification tasks can result in decreased recognition capability for the less numerous classes, thereby affecting the overall performance of the model. In this study, both the training and testing cohorts exhibited an imbalance in sample categories, with a DCB to NDB ratio of approximately 2.4:1. As a result, during model training, the algorithm may have developed a bias favoring the identification of DCB patients, which in turn led to reduced performance in recognizing NDB patients. Ultimately, this bias contributed to a decrease in AUC(PR) within the testing cohort.

Additionally, we performed dynamic longitudinal imaging analysis and found that the COMB-Radscore has the potential to become a dynamic biomarker that can guide subsequent immunotherapy. However, it is important to note that only 25 patients underwent dynamic longitudinal imaging analysis because ^18^F-FDG PET/CT is not commonly used in clinical follow-up. Therefore, the effectiveness of the COMB-Radscore in this clinical scenario remains to be prospectively validated.

Tumor heterogeneity is a prevalent and significant characteristic in the processes of tumor initiation and progression. Hong et al. conducted an analysis of PD-L1 expression in 1,398 NSCLC patients to examine its heterogeneity and its effects on the efficacy of immune checkpoint inhibitor (ICI) therapy [9]. In this study, we evaluated the predictive value of PD-L1 expression across various biopsy sites for immunotherapy in NSCLC patients. Our findings indicated that PD-L1 expression in lung biopsy sites demonstrated optimal performance in predicting the outcomes of ICI therapy. However, we observed no significant association between PD-L1 expression in other metastatic biopsy sites and patient responses to ICI therapy or overall survival. This finding is not exactly identical to the conclusions drawn by Hong et al., which may be attributed to the significant differences in the composition of the metastatic sites within our study cohort (n = 18; eight from the pleura, four from the brain, three from muscle, three from bone, and three from the liver). Furthermore, significant heterogeneity was observed in the radiomic features of primary lung tumors, liver metastases, and adrenal metastases. This reminds us that extending models based on single-organ radiomic features to other metastatic tumors remains a significant challenge.

Single-modal data do not fully describe the status of the cancer. The integration of multi-modal data, such as tissue pathology, radiology, genomics, and clinical information, is expected to further advance the development of precision oncology [25]. In this study, we have integrated the data from the COMB-Radscore and TPS-Lung to develop an integrated model called TPS-Radscore, which has further improved prediction performance.

The data-driven radiomics approach essentially fails to elucidate the underlying biological mechanisms [26]. The disconnect between radiomic models and their biological implications greatly limits their widespread clinical application. Therefore, the relationship between radiomics and biological significance has garnered growing attention from researchers. Sun et al. developed a radiomics score for tumor-infiltrating CD8+ cells, which was correlated with tumor immunophenotype, pathology, and clinical outcomes [27].

In this study, we conducted radiogenomic analysis, and the observed results demonstrate that COMB-Radscore is associated with the abundance and functional status of CD8 + T cells, which potentially elucidates the underlying mechanism for the accurate prediction of immunotherapy efficacy in NSCLC patients by COMB-Radscore. However, further validation through additional foundational experiments is necessary to verify these conclusions.

There were some limitations in our research. First, the patient data were obtained from a single-center cohort, with the majority of patients hailing from a specific geographic region in China. The distribution of their clinical and pathological characteristics may differ from global trends, and the generalizability of the model should be further validated through external verification. Second, this study was retrospective; therefore, the model may be affected by selection bias. In addition, the PFS and OS follow-up data we collected inevitably contain right censoring, which is particularly serious in the OS data. This may lead to an underestimation of the true survival rate of patients. In this study, RECIST 1.1 was employed to evaluate the efficacy of immunotherapy. However, it is essential to consider iRECIST for assessing immunotherapy outcomes, as it incorporates novel response patterns to ICI, including pseudoprogression and hyperprogression, despite its limited adoption in clinical practice.

## Conclusions

In summary, our research involved developing and validating a machine-learning model based on intratumoral and peritumoral ^18^F-FDG PET/CT radiomics. The model demonstrates excellent predictive ability in forecasting the response of NSCLC patients to immunotherapy, which may provide precise, personalized, and dynamic decision support for the treatment of NSCLC patients. However, it should be noted that these findings require further validation through prospective studies.

## Supplementary information

Supplementary Results

Supplementary Methods

Supplementary Figures

Supplementary Tables

Supplementary Material(Original ROC Curves)

## Data Availability

The data of the external radiogenomic cohort used and analyzed in this study can be accessed in the TCIA database, specifically in the TCGA-LUAD (10.7937/K9/TCIA.2016.JGNIHEP5) and TCGA-LUSCdatasets (10.7937/K9/TCIA.2016.TYGKKFMQ). In addition, the remaining data and computer codes that support the findings of this study can be obtained from the corresponding authors upon reasonable request.
