# Supplementary material for: Intratumoral and peritumoral PET/CT-based radiomics for non-invasively and dynamically predicting immunotherapy response in NSCLC
Source: Br J Cancer. 2025 Feb 10;132(6):558–68. doi: 10.1038/s41416-025-02948-z (PMC11920075; doi:10.1038/s41416-025-02948-z)
Supplement: Supplementary file 1 — Supplementary Results [file 41416_2025_2948_MOESM1_ESM.docx]

**Supplementary Results**

**(1). Spatial Heterogeneity of COMB-Radscore and PD-L1 (TPS)**

The patients were divided into the following three cohorts based on biopsy site: lung (Lung, n = 182), lymph node metastases (LN, n = 58), and other organ metastases (Metastasis, n = 21). TPS-Lung exhibited superior performance in predicting immunotherapy response, achieving an AUC (ROC) of 0.721 (p-value < 0.0001) (Figure S6A). Compared to NDB patients, DCB patients demonstrated elevated levels of TPS-Lung levels (Figure S6B). However, the predictive ability of TPS-LN and TPS-Metastasis was found to be inadequate, with AUC (ROC) of 0.511 (p-value = 0.8077) and 0.235 (p-value = 1.0000), respectively (Figure S6A). There was no statistically significant difference in the TPS-LN and TPS-Metastasis levels between the DCB and NDB groups (Figure S6B). Survival analysis further confirmed that only TPS-Lung possessed stratifying capacity for PFS (log-rank test p-value = 0.021) and OS (log-rank test p-value = 0.059) (Figure S6C–E).

We then conducted analysis to investigate the presence of spatial heterogeneity in the prediction capacity of the COMB-Radscore. A total of 28 patients with liver metastases and 35 patients with adrenal metastases were selected from the training and testing cohorts. Following the same radiomic workflow, we derived the COMB-Radscore based on lesions from the liver and adrenal metastases. The predictive performance of COMB-Radscore (Liver) significantly decreased, yielding an AUC (ROC) of 0.651 (p-value = 0.1782) (Figure S7A). Moreover, there was no longer a statistically significant difference in the distribution of the COMB-Radscore (Liver) between the two patient groups, and its stratification capacity for PFS and OS had also diminished (Figure S7B–D).

The predictive performance of the COMB-Radscore (Adrenal) was significantly inferior, with an AUC (ROC) of 0.363 (p-value = 1.0000) (Figure S8A). Likewise, there was no difference between the two groups of patients in terms of COMB-Radscore (Adrenal) (Figure S8B). It is worth noting that survival analysis indicated that the results of stratification based on COMB-Radscore (Adrenal) were opposite to those of COMB-Radscore (Lung) (Figure S8C and D).

Subsequently, we conducted further analysis to assess the consistency and correlation of the radiomic features of the COMB-Radscore model between primary lung tumors and liver metastases, as well as between primary lung tumors and adrenal metastases (Table S12–13). The findings revealed a lack of significant positive consistency or correlation among most radiomic features across these anatomical locations.

**(2). COMB-Radscore as a Biomarker for Differentiating Immunotherapy Strategies in NSCLC Patients with TPS < 50%**

It is well established that both immune monotherapy and immune combination chemotherapy have been approved for NSCLC patients with a TPS ≥ 50%. However, in the population of NSCLC with a TPS < 50%, there are currently no recognized biomarkers to differentiate the patient population that would benefit from immunotherapy monotherapy or combination therapy. Therefore, we further evaluated the potential application value of COMB-Radscore in this clinical scenario. Survival analysis revealed that patients in the low COMB-Radscore group did not show a significant improvement in PFS with immune combination therapy compared to immunotherapy monotherapy (log-rank test p-value = 0.224) (Figure S11). Upon careful observation, we noted that the PFS decline was more rapid in the first few months for patients receiving immunotherapy monotherapy compared to those receiving immunotherapy combination therapy; however, these two curves gradually overlapped over time. This suggests that for patients with a TPS < 50% and a low COMB-Radscore, immunotherapy combination therapy may be superior to immunotherapy monotherapy in delaying early progression but does not confer long-term PFS benefits. Conversely, patients with a high COMB-Radscore who received combined immunotherapies achieved a significant improvement in PFS (log-rank test p-value = 0.017). These findings indicate that immune monotherapy was not inferior to combination immunotherapies for NSCLC patients with a TPS < 50% and a low COMB-Radscore. However, more aggressive combination therapies may be required for those with a TPS < 50% and a high COMB-Radscore.
