# Supplementary material for: Intratumoral and peritumoral PET/CT-based radiomics for non-invasively and dynamically predicting immunotherapy response in NSCLC
Source: Br J Cancer. 2025 Feb 10;132(6):558–68. doi: 10.1038/s41416-025-02948-z (PMC11920075; doi:10.1038/s41416-025-02948-z)
Supplement: Supplementary file 2 — Supplementary Methods [file 41416_2025_2948_MOESM2_ESM.docx]

**Supplementary** **Methods**

**(1). Clinicopathological Characteristics and Laboratory Test Data Collection**

We collected patient clinical and pathological characteristics as well as laboratory examination data at baseline. This included gender, age, body mass index (BMI), smoking status, histological type, TNM stage, metastases number, treatment strategy, local radiotherapy, occurrence of immune-related adverse events (irAE), PDL1 (TPS), tumor markers, complete blood count, albumin (ALB), C-reactive protein (CRP), and lactate dehydrogenase (LDH).

Furthermore, we calculated the levels of serum inflammatory markers that were previously proven to be associated with immunotherapy including neutrophil-to-lymphocyte ratio (NLR), derived neutrophil-to-lymphocyte ratio (dNLR), lymphocyte-to-monocyte ratio (LMR), platelet-to-lymphocyte ratio (PLR), systemic immune-inflammation index (SII), prognostic nutritional index (PNI), advanced lung cancer inflammation index (ALI), lung immune prognostic index (LIPI), Glasgow prognostic score (GPS), and modified Glasgow prognostic score (mGPS)^[1-9]^.

**(2). ^18^F-FDG PET/CT Image Acquisition**

Before treatment, all patients were examined with a whole-body fluoro-18-fluorodeoxyglucose (^18^F-FDG) positron emission tomography/computed tomography (PET/CT) using either a Biograph mCTx scanner (Siemens Healthcare) or a uEXPLORER total-body PET/CT scanner (United Imaging Healthcare). ^18^F-FDG was manufactured automatically using a tracer synthesis system (TRACERlabFX2N, GE Healthcare) and the ^18^F-FDG synthesizer module Tracerlab FXF-N (Beijing PET Biotechnology Co. Ltd). The radiochemical purity of ^18^F-FDG exceeded 95%, and all radiotracers were sterile and pyrogen-free to meet the set criteria for human administration.

First, patients were instructed to fast for at least 6 h prior to ^18^F-FDG PET/CT scanning. The patient's fasting blood glucose was monitored using a blood glucose meter to ensure that the blood glucose level was < 11.1 mmol/L before the ^18^F-FDG injection. Then, patients were injected with 270–410 MBq of ^18^F-FDG according to body weight (~ 5.55 MBq/kg), and PET/CT scanning was performed after 60-min of uptake. All patients kept their arms up and maintained shallow breathing to reduce artifacts during acquisition.

The acquired CT data were reconstructed using a standard lung reconstruction algorithm (parameters: in-plane resolution 0.82 × 0.82 mm^2^, slice thickness 1.5 mm, slice spacing 1 mm, convolution kernel B70f). The acquired PET data were reconstructed using an ordered-subset expectation maximization (OSEM) algorithm (3 iterations, 21 subsets), and attenuation correction and scatter correction were performed using the CT transmission data (parameters: in-plane resolution 4.07 × 4.07 mm^2^; slice thickness 5 mm; slice spacing 3 mm). Then, to eliminate quantitative differences between patient size and ^18^F-FDG injection dose, the intensity value of the PET images was converted to a standardized uptake value (SUV) by normalizing the injection dose and patient weight for subsequent analysis.

**(3). Image Fusion**

Single-modality CT or single-modality PET images cannot fully elucidate the complex heterogeneity of tumors. Therefore, to integrate the advantages of CT for anatomical localization and PET for functional metabolism, we fused PET and CT images into PET/CT images.

Methodology for image fusion: I. Resampling of PET images was carried out to the same voxel spacing as the CT images using linear interpolation (0.82 × 0.82 × 1.0 mm^3^). II. Normalization of the intensity range of the PET and CT images between 0 and 255 was then done. III. PET images were registered to the CT images using the image registration tool Elastix.^[10]^ IV. PET and CT images were fused using a specific ITK filter (ConstrainedValueAdditionImageFilter) ^[11]^ to obtain PET/CT images.

**(4). Image Segmentation**

We used the image registration tool Elastix to register the PET image to the CT image (PET/CT image was also registered to the CT image before fusion) to ensure that the spatial position, size, and shape of the primary tumor were essentially the same in all images before image segmentation. In addition, to reduce potential segmentation errors and ensure that the volume of interest (VOI) in the single-modality images (CT and PET) and multi-modality images (PET/CT) remained constant, we chose to manually draw the VOI layer by layer along the contour of the primary tumor on the PET/CT images and apply it to all images.

We partially adopted the multi-VOI segmentation strategy proposed by Lin et al. (2022)^[12]^ for image segmentation to comprehensively characterize tumors: I. Tumoral: an experienced physician (Reader 1) manually drew the VOI layer by layer along the contour of the primary tumor on the PET/CT images. In the case of more than one tumor foci, the largest tumor focus was segmented. II. Peritumoral: the peritumoral area was obtained by expanding the tumor outward using a width of 15-mm and subtracting the tumor area (expanding operations were implemented automatically based on dilating algorithms, with a sphere morphological structuring element (radius = 15 mm)). The selection of the size of the peritumoral region was dependent on the research findings of Mohiuddin et al. (2014),^[13]^ which revealed that surgical resection margins larger than 15 mm around the lung lesion do not provide additional benefit in terms of disease recurrence. III. Intratumoral: the intratumoral area was obtained by shrinking by a 5-mm width (shrinking operations were implemented automatically based on eroding algorithms, with a sphere morphological structuring element (radius = 5 mm)). IV. Tumor-Peritumoral: tumor and peritumoral regions were integrated to form the tumor-peritumoral region.

After 1 month, we explored the reproducibility of the image features by randomly selecting 80 patients and repeating the image segmentation by Reader 1 and Reader 2, using the intra-/inter-class correlation coefficient (ICC). In this way, we were able to assess the intra-/inter-observer consistency of features. Image segmentation was done using 3Dslicer (version 5.0.4, https://www.slicer.org/) and was supervised by senior nuclear medicine specialists.

**(5). Image Preprocessing and Feature Extraction**

We adopted three steps to preprocess images prior to feature extraction. First, to minimize the influence of different layer thicknesses, we resampled all images to the same isotropic voxel spacing (1.0 × 1.0 × 1.0mm^3^) by B-spline interpolation. Second, based on the gray-scale discretization process (bin count for PET = 32, bin width for CT and PET/CT = 25), we converted the continuous image into discrete integer values. Finally, images were processed by eight methods, namely, Square, Square root, Logarithm, Exponential, Gradient, Local Binary Pattern (LBP), Laplacian of Gaussian (LoG), and Wavelet filtering. Using the line with the imaging biomarker standardization initiative, radiomic features extraction from PET, CT, and PET/CT images was performed via PyRadiomics ^[14]^(version 3.0.1, https://github.com/Radiomics/pyradiomics) in Python (version 3.7.0, <https://www.python.org/>). PET, CT, and PET/CT each capture distinct information pertaining to lesions. As such, we posit that the radiomic features extracted from these images likewise encompass varied information about the lesions, potentially offering insights into treatment efficacy or prognosis across different dimensions. The PyRadiomics parameter settings for image preprocessing and feature extraction are described in Table S16. The comprehensive radiomic features list is presented in Table S17.

**(6). Feature Selection**

Due to the large variation in values between radiomic features, we used the Z-score method to standardize the features before making feature selections to reduce the influence of different magnitudes between features. In addition, to build a more robust model, we specifically selected image features with intra-/inter-class ICC values > 0.75 for subsequent analysis.

To avoid dimensionality disasters and overcome the redundancy of radiomic features, we followed a strict feature selection process in the training cohort. I. Student's *t* test was used to detect whether the means of the features in the independent samples of the two groups (DCB and NDB) showed a significant difference, retaining the features with a *p*-value < 0.05. II. The Minimum Redundancy Maximum Relevance (mRMR) algorithm was used to identify features that were highly correlated with efficacy but minimally related to other features, and the features of the top 50 were retained. III. The 10-fold cross validation and the Least Absolute Shrinkage and Selection Operator (LASSO) method were used to retain robust and non-redundant features. IV. The 10-fold cross-validation and the Recursive Feature Elimination (RFE) algorithm were used to retain the optimal subset of features.

**(7). Model Evaluation**

The discrimination ability of each model was evaluated by plotting the receiver operating characteristic (ROC) curves and calculating the area under the curve (AUC). The "binormal" method was employed to smooth the curves. This technique utilizes a binormal distribution model to fit the original data points, effectively smoothing the curves while preserving their authenticity in representing model performance. Additionally，all the original ROC curves of this study are provided in the supplementary material (Original ROC curves). The calibration curve and the Hosmer–Lemeshow goodness-of-fit test were used to assess the degree of deviation between the predicted values and the actual values in the model. Decision curve analysis was used to evaluate the clinical utility of the model. Precision-recall (PR) curves were generated, and the AUC was calculated to evaluate the predictive performance of the model on specific sample classes (NDB) while considering the imbalance in the distribution of sample classes. The 95% confidence interval of the AUC value of the ROC and PR curves was determined using the bootstrap method with 2,000 iterations. The net reclassification improvement (NRI) and integrated discrimination improvement (IDI) were used to compare the predictive abilities of two models, allowing for a quantitative evaluation of the improvement in the predictive ability of one model over another. In addition, several evaluation metrics, including positive predictive value (PPV), negative predictive value (NPV), sensitivity, specificity, accuracy (ACC), recall, F1 score, Matthews correlation coefficient (MCC), and Kappa, were calculated.

**(8). Validation of Dynamic Predictive Capabilities**

The same radiomic workflow, which includes image fusion, image segmentation, image preprocessing, and feature extraction, was applied to the follow-up ^18^F-FDG PET/CT scans of 25 patients. Quantitative radiomic features were extracted from a total of 12 VOIs across three imaging modalities: PET, CT, and PET/CT.

Subsequently, the radiomic features were incorporated into the established model, COMB-Radscore, to derive each patient's follow-up COMB-Radscore. Finally, the predictive performance of the follow-up COMB-Radscore in dynamically forecasting subsequent treatment outcomes and prognosis was assessed.

**(9). Gene set enrichment analysis**

In order to investigate the biological basis of radiomics score, gene set enrichment analysis (GSEA)^[15]^ (www.gsea-msigdb.org/gsea/index.jsp) was performed. GSEA was conducted using clusterProfiler package^[16]^ (version 4.1). The annotated gene sets (Gene ontology: biological process (GO: BP), Kyoto encyclopedia of genes and genomes (KEGG) and Hallmark) downloaded from Molecular Signatures Database (MSigDB) were selected as the reference gene sets. The FDR (q-value) < 0.25 and p < 0.05 were considered statistically significant.

**(10). Evaluation of the immune microenvironment**

We analyzed the differences in the immune microenvironment between high and low imaging score patients using Immuno-Oncology Biological Research (IOBR) ^[17]^, an immunology tool previously developed by our research group. The immunophenoscore (IPS)^[18]^ was used to calculate the scores of four different immune phenotypes (MHC molecules, effector cells, suppressor cells, checkpoints) for each patient. Cibersort^[19]^ was used to evaluate the infiltration abundance of immune cells in patients. Two gene markers, T-cell–inflamed gene-expression profile (GEP)^[20]^ and cytolytic activity(CYT)^[21]^, were used to analyze the T cell functional status of the two groups of patients.

**(11). Multiplexed quantitative immunofluorescence**

Multiple immunofluorescence staining was performed on the pathological slides of NSCLC patients before treatment to quantify tumor-infiltrating T cells of different phenotypes. 4 μm thick sections were cut from formalin-fixed paraffin-embedded (FFPE) NSCLC patients lung tissues blocks. The slides were dewaxed and antigen-repaired with the one-step dewaxing/antigen retrieval buffer (pH9.0) in Quadruple-Fluorescence immunohistochemical mouse/rabbit kit (immunoway, RS0037) and the endogenous peroxidase was blocked by peroxidase blocking buffer. The sections were incubated overnight at 4 °C with the primary antibodies against CD8 (Abcam, ab93278, 1:200) at the first day.

The next day, after rewarming at room temperature for 30 min, Horseradish Peroxidase (HRP) -labeled goat anti-mouse or anti-rabbit secondary antibody were stained for 1h at room temperature, protected from light. After washing out the secondary antibody, use 594-labeled Tyramide dye for a 10 min color reaction. Then after antibody stripping was carried out using the Antibody Stripping Solution, we successively labeled PD1 with 488-labeled Tyramide (Servicebio, gb14131, 1:100), PRF1 with 525-labeled Tyramide (Immunoway, YT5792, 1:100), CD3 with 647-labeled Tyramide (Abcam, ab16669, 1:150) following previously described labeling steps of CD8. Finally, sections were counterstained with DAPI for 10 min and sealed with the anti-fluorescence quencher.

Samples were scanned using Multidimensional Panoramic Tissue Scanner (TissueGnostics, TissueFAXS), and representative multiplex immunofluorescence images are shown, manually removing the images with necrotic tissue or staining artifacts. Image analysis software (TissueGnostics, StrataQuest7.1) was used to process images, segment cells and define specific phenotypes. 2-6 High-powered microscopic fields (400x; 0.0625 mm2) per slide were selected to calculate the density of CD3+CD8+, CD3+CD8+PRF1+, CD3+CD8+PDL1+, CD3+CD8+PRF1+PDL1+ cells in all nucleated cells of the tumor nests and tumor stroma, with their averages calculated. Two researchers independently evaluated the stained slides.

**References:**

[1]. Bagley SJ, Kothari S, Aggarwal C, Bauml JM, Alley EW, Evans TL et al. Pretreatment neutrophil-to-lymphocyte ratio as a marker of outcomes in nivolumab-treated patients with advanced non-small-cell lung cancer. *Lung Cancer*. 2017;106:1-7

[2]. Alessi JV, Ricciuti B, Alden SL, Bertram AA, Lin JJ, Sakhi M et al. Low peripheral blood derived neutrophil-to-lymphocyte ratio (dNLR) is associated with increased tumor T-cell infiltration and favorable outcomes to first-line pembrolizumab in non-small cell lung cancer. *J Immunother Cancer*. 2021;9(11)

[3]. Prelaj A, Rebuzzi SE, Pizzutilo P, Bilancia M, Montrone M, Pesola F et al. EPSILoN: A Prognostic Score Using Clinical and Blood Biomarkers in Advanced Non-Small-cell Lung Cancer Treated With Immunotherapy. *Clin Lung Cancer*. 2020;21(4):365-377.e5

[4]. Diem S, Schmid S, Krapf M, Flatz L, Born D, Jochum W et al. Neutrophil-to-Lymphocyte ratio (NLR) and Platelet-to-Lymphocyte ratio (PLR) as prognostic markers in patients with non-small cell lung cancer (NSCLC) treated with nivolumab. *Lung Cancer*. 2017;111:176-181

[5]. Banna GL, Cantale O, Muthuramalingam S, Cave J, Comins C, Cortellini A et al. Efficacy outcomes and prognostic factors from real-world patients with advanced non-small-cell lung cancer treated with first-line chemoimmunotherapy: The Spinnaker retrospective study. *Int Immunopharmacol*. 2022;110:108985

[6]. Johannet P, Sawyers A, Qian Y, Kozloff S, Gulati N, Donnelly D et al. Baseline prognostic nutritional index and changes in pretreatment body mass index associate with immunotherapy response in patients with advanced cancer. *J Immunother Cancer*. 2020;8(2)

[7]. Mountzios G, Samantas E, Senghas K, Zervas E, Krisam J, Samitas K et al. Association of the advanced lung cancer inflammation index (ALI) with immune checkpoint inhibitor efficacy in patients with advanced non-small-cell lung cancer. *ESMO Open*. 2021;6(5):100254

[8]. Mezquita L, Auclin E, Ferrara R, Charrier M, Remon J, Planchard D et al. Association of the Lung Immune Prognostic Index With Immune Checkpoint Inhibitor Outcomes in Patients With Advanced Non-Small Cell Lung Cancer. *JAMA Oncol*. 2018;4(3):351-357

[9]. Takamori S, Takada K, Shimokawa M, Matsubara T, Fujishita T, Ito K et al. Clinical utility of pretreatment Glasgow prognostic score in non-small-cell lung cancer patients treated with immune checkpoint inhibitors. *Lung Cancer*. 2021;152:27-33

[10]. Klein S, Staring M, Murphy K, Viergever MA, Pluim JP. elastix: a toolbox for intensity-based medical image registration. *IEEE Trans Med Imaging*. 2010;29(1):196-205

[11]. McCormick M, Liu X, Jomier J, Marion C, Ibanez L. ITK: enabling reproducible research and open science. *Front Neuroinform*. 2014;8:13

[12]. Jiang L, You C, Xiao Y, Wang H, Su GH, Xia BQ et al. Radiogenomic analysis reveals tumor heterogeneity of triple-negative breast cancer. *Cell Rep Med*. 2022;3(7):100694

[13]. Mohiuddin K, Haneuse S, Sofer T, Gill R, Jaklitsch MT, Colson YL et al. Relationship between margin distance and local recurrence among patients undergoing wedge resection for small (≤2 cm) non-small cell lung cancer. *J Thorac Cardiovasc Surg*. 2014;147(4):1169-75; discussion 1175-7

[14]. van Griethuysen J, Fedorov A, Parmar C, Hosny A, Aucoin N, Narayan V et al. Computational Radiomics System to Decode the Radiographic Phenotype. *Cancer Res*. 2017;77(21):e104-e107

[15]. Subramanian A, Tamayo P, Mootha VK, Mukherjee S, Ebert BL, Gillette MA et al. Gene set enrichment analysis: a knowledge-based approach for interpreting genome-wide expression profiles. *Proc Natl Acad Sci U S A*. 2005;102(43):15545-50

[16]. Yu G, Wang LG, Han Y, He QY. clusterProfiler: an R package for comparing biological themes among gene clusters. *OMICS*. 2012;16(5):284-7

[17]. Zeng D, Ye Z, Shen R, Yu G, Wu J, Xiong Y et al. IOBR: Multi-Omics Immuno-Oncology Biological Research to Decode Tumor Microenvironment and Signatures. *Front Immunol*. 2021;12:687975

[18]. Charoentong P, Finotello F, Angelova M, Mayer C, Efremova M, Rieder D et al. Pan-cancer Immunogenomic Analyses Reveal Genotype-Immunophenotype Relationships and Predictors of Response to Checkpoint Blockade. *Cell Rep*. 2017;18(1):248-262

[19]. Newman AM, Liu CL, Green MR, Gentles AJ, Feng W, Xu Y et al. Robust enumeration of cell subsets from tissue expression profiles. *Nat Methods*. 2015;12(5):453-7

[20]. Ott PA, Bang YJ, Piha-Paul SA, Razak ARA, Bennouna J, Soria JC et al. T-Cell-Inflamed Gene-Expression Profile, Programmed Death Ligand 1 Expression, and Tumor Mutational Burden Predict Efficacy in Patients Treated With Pembrolizumab Across 20 Cancers: KEYNOTE-028. *J Clin Oncol*. 2019;37(4):318-327

[21]. Rooney MS, Shukla SA, Wu CJ, Getz G, Hacohen N. Molecular and genetic properties of tumors associated with local immune cytolytic activity. *Cell*. 2015;160(1-2):48-61
