# Supplementary material for: Intratumoral and peritumoral PET/CT-based radiomics for non-invasively and dynamically predicting immunotherapy response in NSCLC
Source: Br J Cancer. 2025 Feb 10;132(6):558–68. doi: 10.1038/s41416-025-02948-z (PMC11920075; doi:10.1038/s41416-025-02948-z)
Supplement: Supplementary file 3 — Supplementary Figures [file 41416_2025_2948_MOESM3_ESM.docx]

**Supplementary Figures**


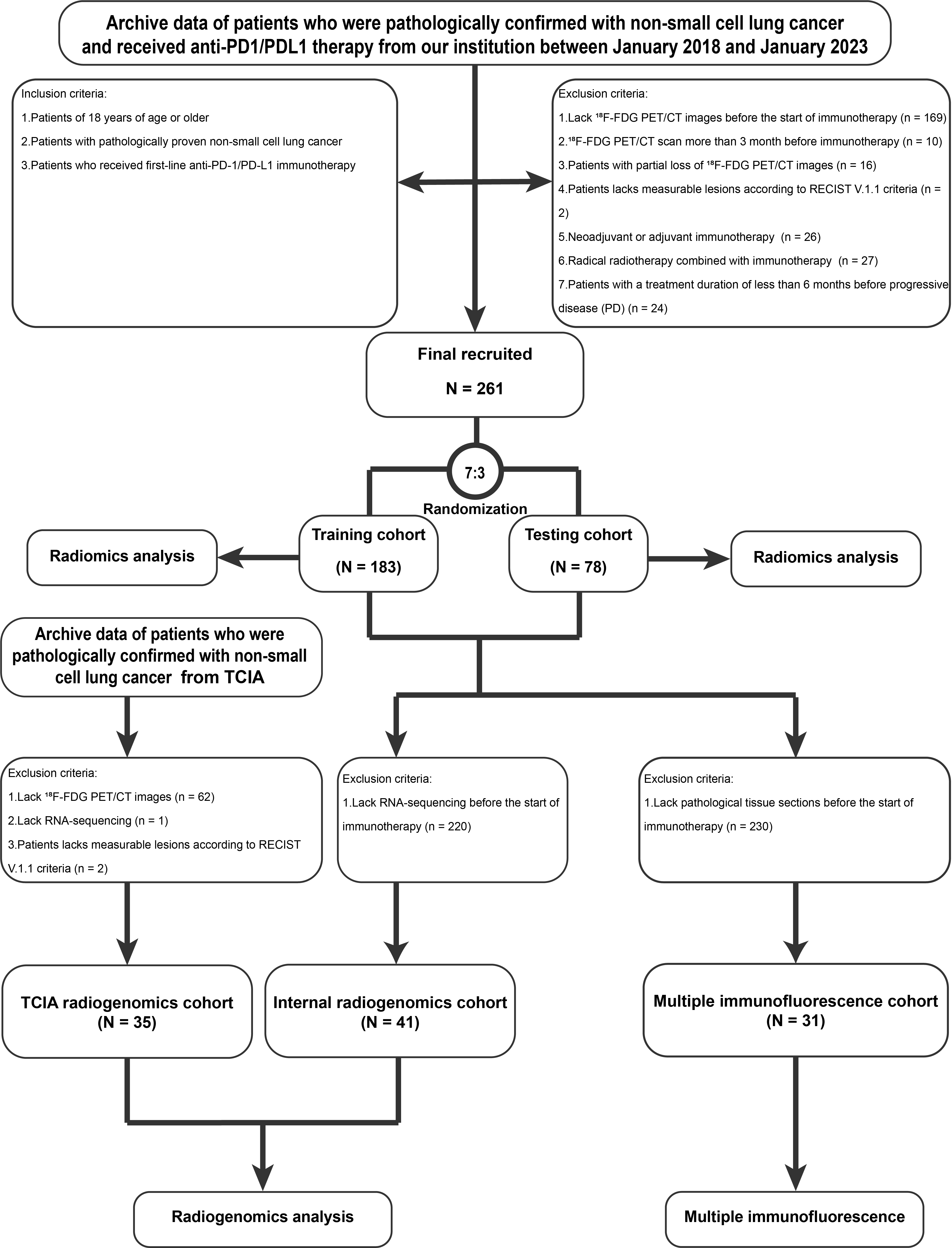


**Figure S1: The detailed flowchart of patient selection**

PD1/PD-L1, programmed death 1/programmed death ligand 1; ^18^F-FDG PET/CT, fluoro-18-fluorodeoxyglucose positron emission tomography/computed tomography; RECIST, response evaluation criteria in solid tumors; PD, progressive disease; TCIA, The Cancer Imaging Archive.

The definitions of PD were based on the RECIST V.1.1 criteria.





**Figure S2: Performance evaluation of prediction models**

**A–D** ROC curves (**A**), calibration curves (**B**), decision curves (**C**), and PR curves (**D**) of the four radiomic models in the training cohort.

**E** Difference in COMB-Radscore between DCB and NDB groups in the training cohort.

**F** Response (DCB/NDB) and COMB-Radscore for each patient in the training cohort.

ROC curves, receiver operating characteristic curves; PR curves, precision-recall curves; AUC, area under the curve; CI, confidence interval; DCB, durable clinical benefit; NDB, no durable clinical benefit.

**
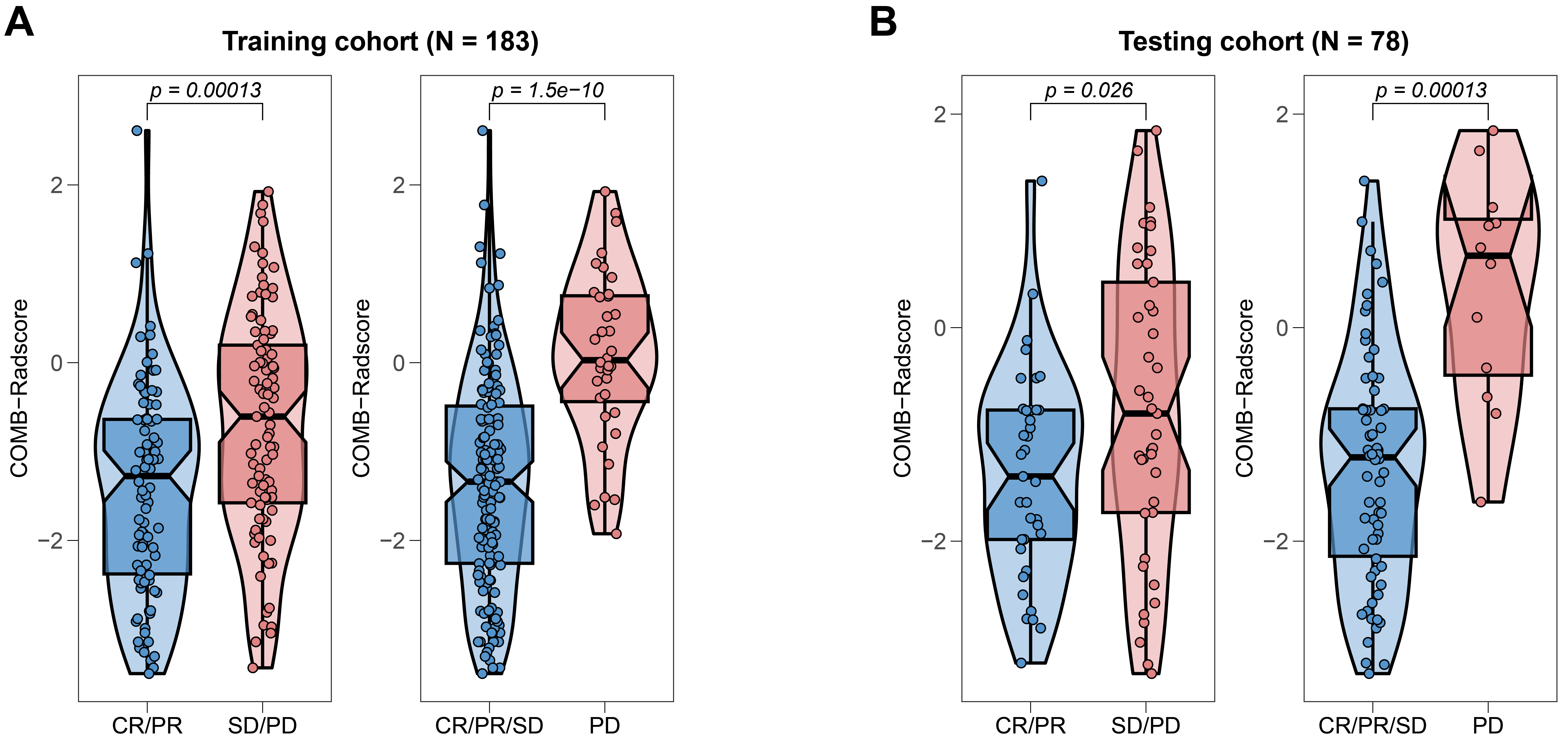
Figure S3: The Difference in COMB-Radscore across different responses groups**

**A, B** The difference in COMB-Radscore across different responses groups (CR/PR and SD/PD, CR/PR/SD and PD) in the training (**A**) and testing (**B**) cohorts.

CR, Complete response; PR, Partial response; SD, Stable disease; PD, Progressive disease

The definitions of CR, PR, SD, and PD were based on the RECIST V.1.1 criteria.





**Figure S4: COMB-Radscore and serum inflammatory markers**

**A, B** ROC curves of 10 serum inflammatory markers and COMB-Radscore in the training (**A**) and testing (**B**) cohorts.

**C** Heatmap of correlation between COMB-Radscore and 10 serum inflammatory markers in training (left) and testing (right) cohorts.

ROC curves, receiver operating characteristic curves; AUC, area under the curve; CI, confidence interval; NLR, neutrophil-to-lymphocyte ratio; dNLR, derived neutrophil-to-lymphocyte ratio; LMR, lymphocyte-to-monocyte ratio; PLR, platelet-to-lymphocyte ratio; SII, systemic immune-inflammation index; PNI, prognostic nutritional index; ALI, advanced lung cancer inflammation index; LIPI, lung immune prognostic index; GPS, Glasgow prognostic score; mGPS, modified Glasgow prognostic score.


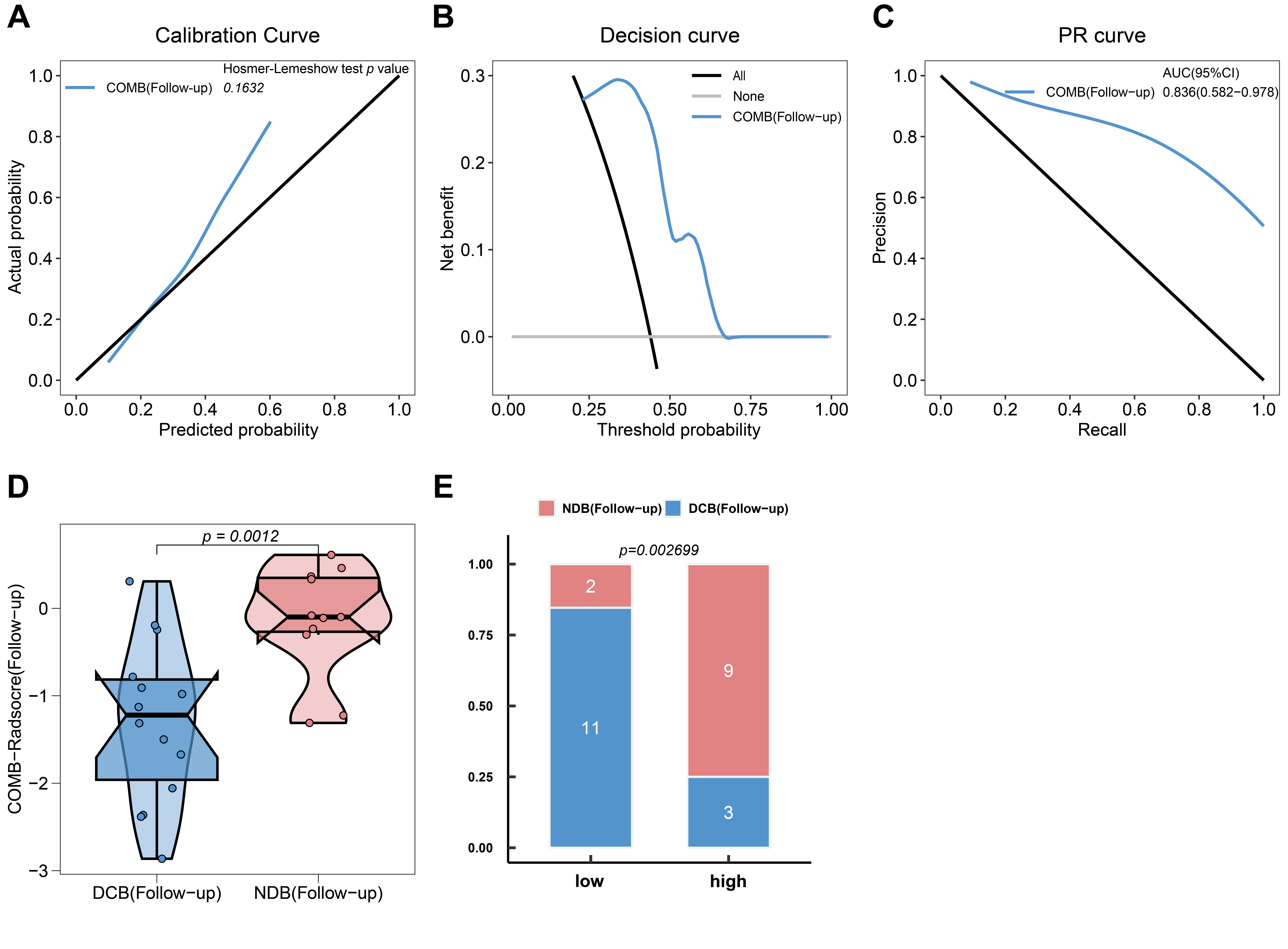


**Figure S5: Dynamic predictive ability of and changes in COMB-Radscore**

**A–C** Calibration curve (**A**), decision curve (**B**) and PR curve (**C**) of COMB-Radscore (Follow-up) in the follow-up cohort.

**D** Difference in COMB-Radscore (Follow-up) between DCB (Follow-up) and NDB (Follow-up) groups in the follow-up cohort.

**E** Proportional composition of different response patients between low and high COMB-Radscore groups in the follow-up cohort.

PR curves, precision-recall curves; AUC, area under the curve; CI, confidence interval; DCB, durable clinical benefit; NDB, no durable clinical benefit.

Based on disease progression within 6 months after follow-up ^18^F-FDG PET/CT, the patients were divided into two groups: the NDB (Follow-up) group consisting of 11 patients with disease progression and the DCB (Follow-up) group consisting of 14 patients without disease progression.





**Figure S6: The spatial heterogeneity of predictive ability of PDL1 (TPS)**

**A** ROC curves of PDL1(TPS) based on different biopsy sites (primary lung tumor, LN metastases and other organ metastases).

**B** Difference of PDL1(TPS) based on different biopsy sites (primary lung tumor, LN metastases and other organ metastases) between DCB and NDB groups.

**C–E** Survival analysis of PFS and OS for low (<50%) and high (≥50%) PDL1 (TPS) groups based on primary lung tumor (**C**), LN metastases (**D**) and other organ metastases (**E**).

ROC curves, receiver operating characteristic curves; AUC, area under the curve; CI, confidence interval; HR, hazard ratio; PDL1(TPS), programmed cell death ligand 1 (tumor proportion score); LN, lymph node; DCB, durable clinical benefit; NDB, no durable clinical benefit; PFS, progression-free survival; OS, overall survival





**Figure S7: The spatial heterogeneity of predictive ability of COMB-Radscore based on liver metastases and primary lung tumor**

**A** ROC curves of COMB-Radscore based on liver metastases and primary lung tumor.

**B** Difference in COMB-Radscore based on liver metastases and primary lung tumor between DCB and NDB groups.

**C, D** Survival analysis of PFS and OS for low and high COMB-Radscore groups based on liver metastases (**C**) and primary lung tumor (**D**).

ROC curves, receiver operating characteristic curves; AUC, area under the curve; CI, confidence interval; HR, hazard ratio; DCB, durable clinical benefit; NDB, no durable clinical benefit; PFS, progression-free survival; OS, overall survival.





**Figure S8: The spatial heterogeneity of prediction ability of COMB-Radscore based on adrenal metastases and primary lung tumor**

**A** ROC curves of COMB-Radscore based on adrenal metastases and primary lung tumor.

**B** Difference in COMB-Radscore based on adrenal metastases and primary lung tumor between DCB and NDB groups.

**C, D** Survival analysis of PFS and OS for low and high COMB-Radscore groups based on adrenal metastases (**C**) and primary lung tumor (**D**).

ROC curves, receiver operating characteristic curves; AUC, area under the curve; CI, confidence interval; HR, hazard ratio; DCB, durable clinical benefit; NDB, no durable clinical benefit; PFS, progression-free survival; OS, overall survival.

**
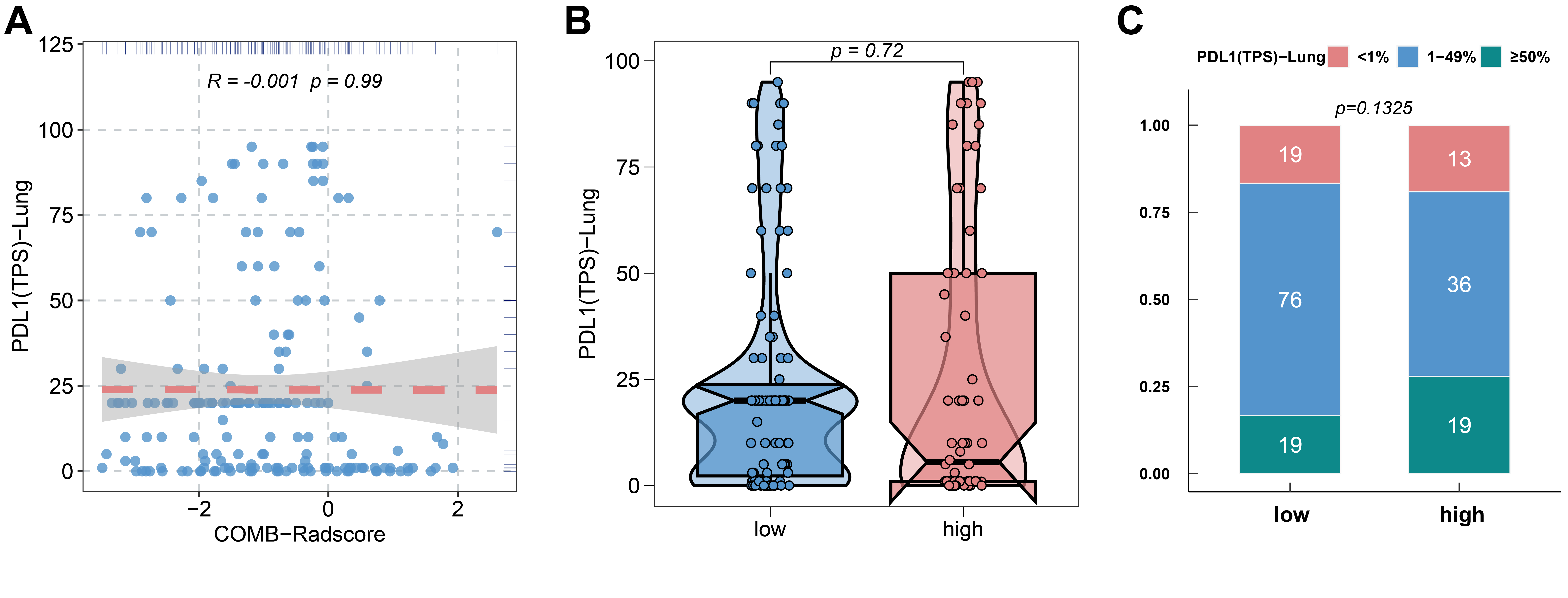
**

**Figure S9: The correlation between COMB-Radscore and PDL1 (TPS)-Lung**

**A** Correlation between COMB-Radscore and PDL1 (TPS)-Lung.

**B** Distribution of PDL1 (TPS)-Lung between low and high COMB-Radscore groups.

**C** Proportional composition of different PDL1 (TPS)-Lung patients between low and high COMB-Radscore groups.

PDL1(TPS), programmed cell death ligand 1 (tumor proportion score).


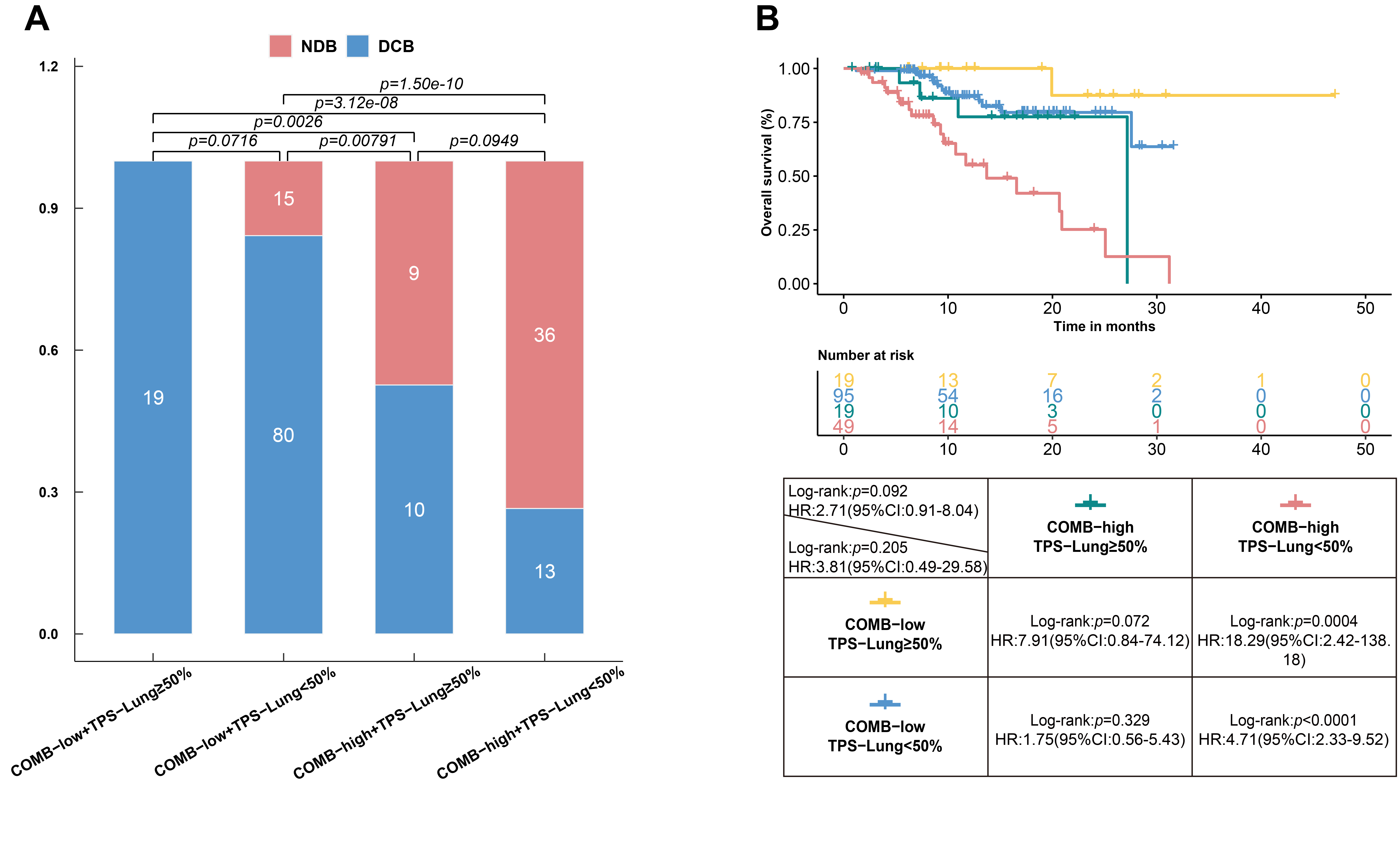


**Figure S10: Combines the COMB-Radscore and TPS-lung to achieve more refined stratification of patients**

**A** Proportional composition of different responses patients in four groups of patients stratified by COMB-Radscore and TPS-Lung.

**B** Kaplan-Meier analysis of OS of four groups of patients stratified by COMB-Radscore and TPS-Lung.

TPS, tumor proportion score; OS, overall survival.


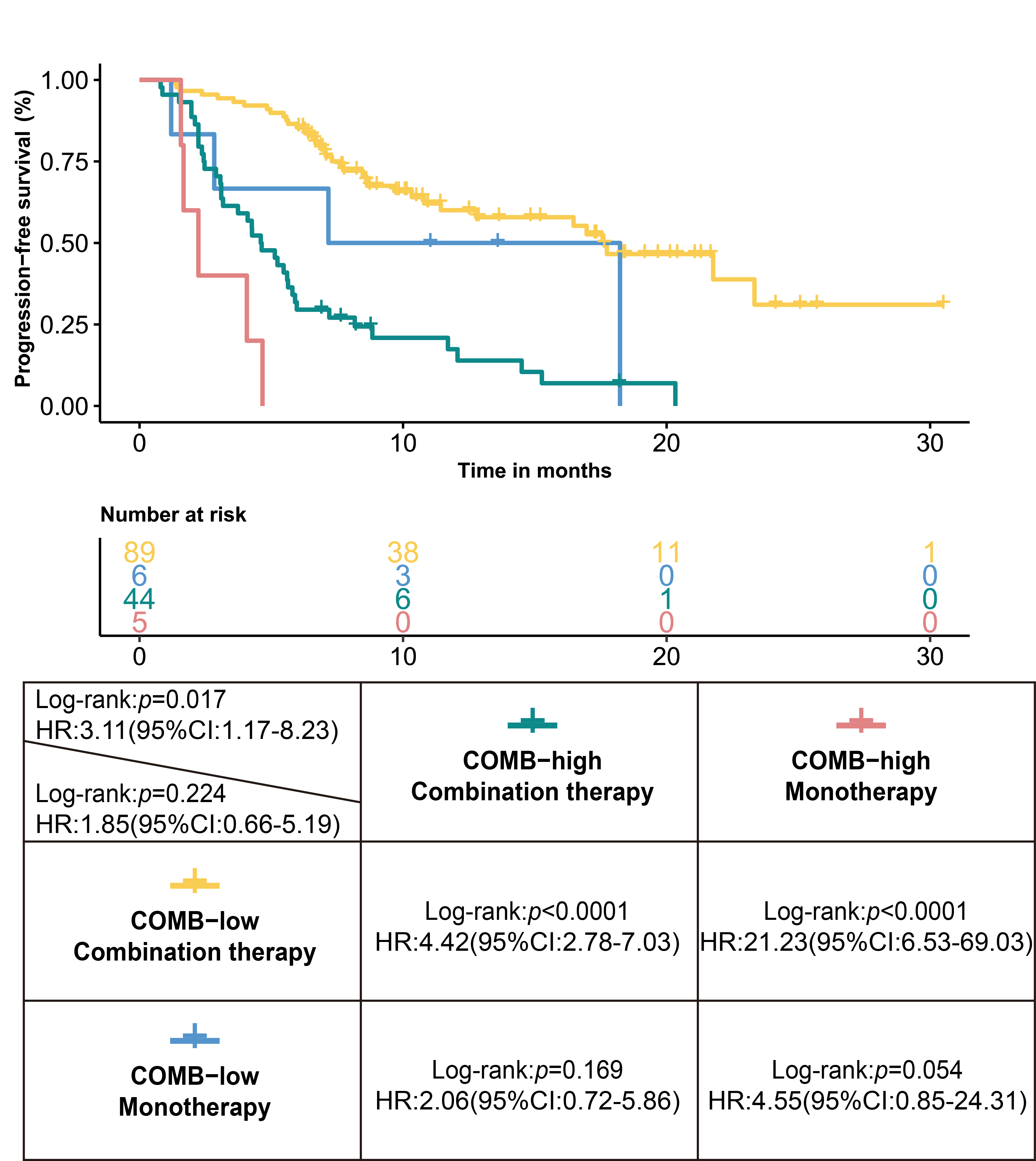


**Figure S11: Potential clinical application of COMB-Radscore for decision-making between PD1/PD-L1 monotherapy and combination therapy in NSCLC patients with TPS <50%**

Combination therapy, combination therapy with immune checkpoint inhibitors, i.e., PD1 (e.g., pembrolizumab or nivolumab)/PD-L1 (e.g., durvalumab) ICI in combination with chemotherapeutic agents (e.g., pemetrexed/paclitaxel/gemcitabine+cisplatin/carboplatin/lobaplatin) and/or antiangiogenic agents (e.g., bevacizumab); Monotherapy, immune checkpoint inhibitor monotherapy, i.e., PD1 (e.g., pembrolizumab or nivolumab)/PD-L1 (e.g., durvalumab) ICI monotherapy; PD1/PD-L1, programmed death 1/programmed death ligand 1; ICI, Immune checkpoint inhibitor.


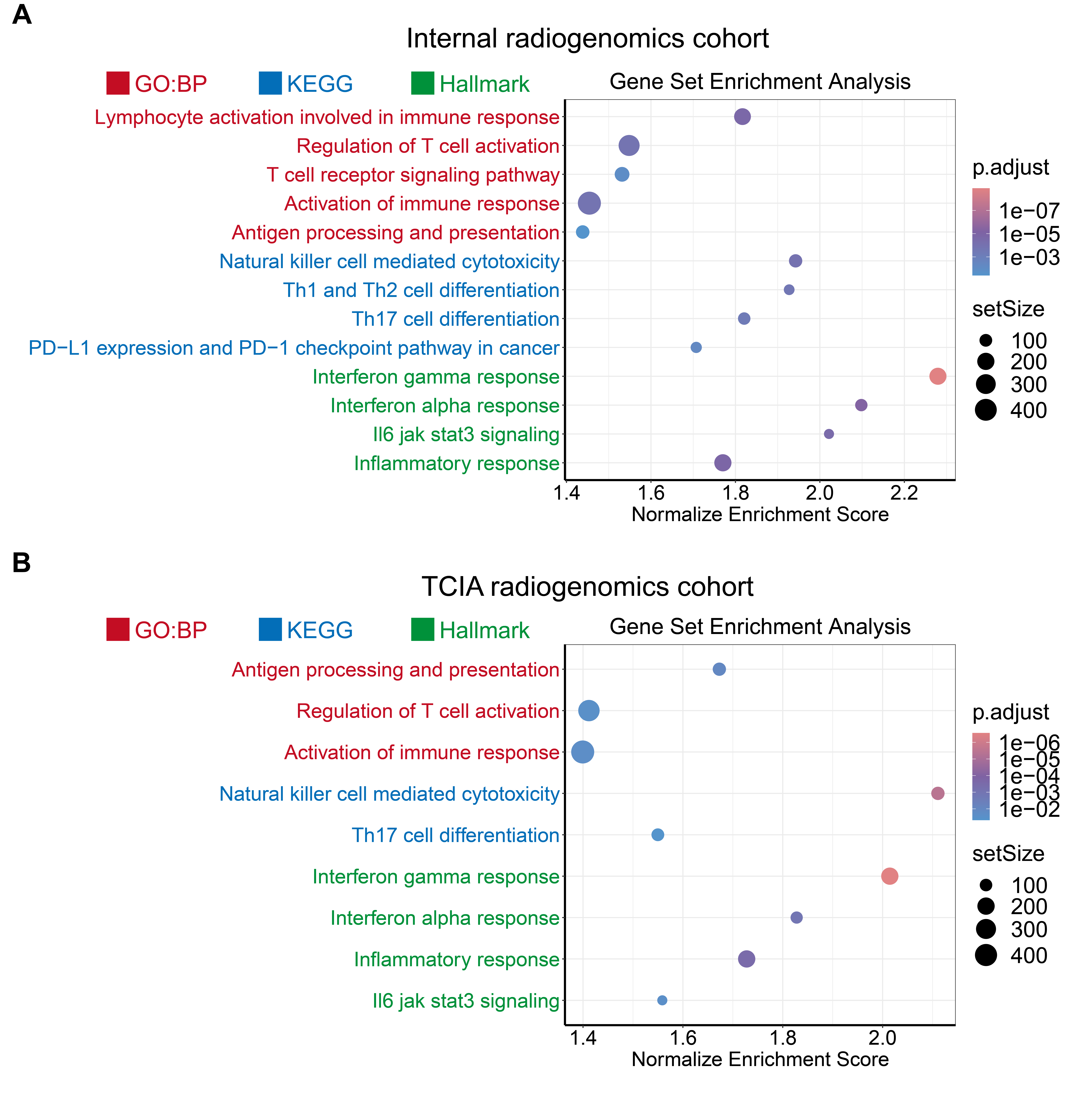


**Figure S12: Gene set enrichment analysis of COMB-Radscore**

**A, B** Gene set enrichment analysis based on the GO: BP, KEGG and Hallmark gene set in the Internal radiogenomics cohort (**A**) and the TCIA radiogenomics cohort (**B**).

GO: BP, gene ontology: biological process; KEGG, kyoto encyclopedia of genes and genomes; TCIA, The Cancer Imaging Archive.


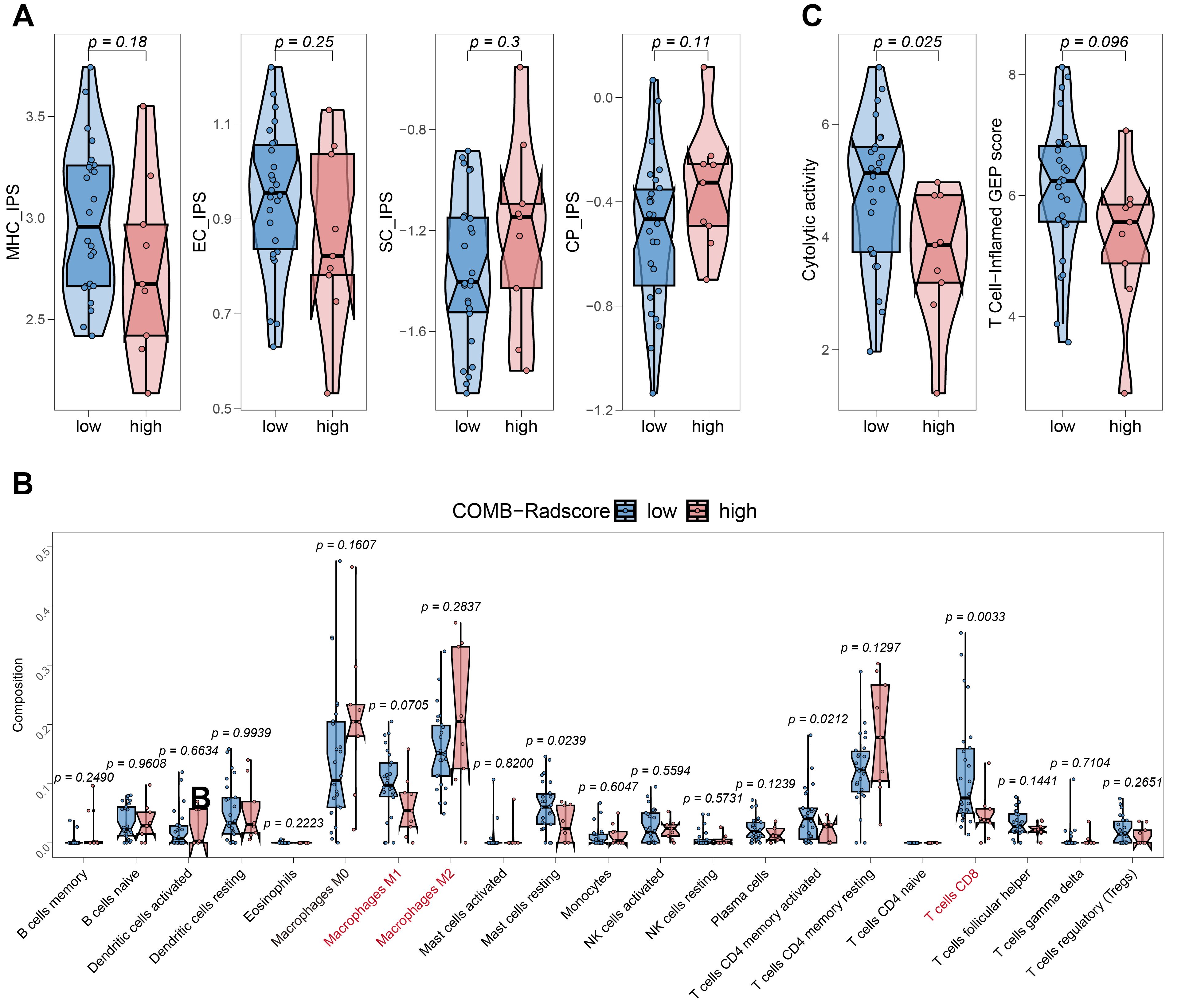


**Figure S13: Differences in the tumor immune microenvironment between the low and high COMB-Radscore groups**

**A-C** Difference in four immune phenotype scores (**A**), abundances of 22 immune cells (**B**), cytolytic activity and T-cell–inflamed GEP score (**C**) between the low and high COMB-Radscore groups in the TCIA radiogenomics cohort.

MHC, major histocompatibility complex; EC, effector cells; SC, suppressor cells, CP, checkpoints; IPS, immunophenoscore; GEP, gene-expression profile; TCIA, The Cancer Imaging Archive.





**Figure S14: Correlation between COMB-Radscore and GZMA, PRF1, PDCD1**

**A, B** Difference in GZMA, PRF1, and PDCD1 gene expression levels between the low and high COMB-Radscore groups in the Internal radiogenomics cohort (**A**) and the TCIA radiogenomics cohort (**B**).

**C, D** Correlation analysis of COMB-Radscore with GZMA, PRF1 and PDCD1 genes in the Internal radiogenomics cohort (**C**) and the TCIA radiogenomics cohort (**D**).

GZMA, granzyme A; PRF1, perforin-1; PDCD1, programmed cell death 1; TCIA, The Cancer Imaging Archive.
