# Supplementary material for: Intratumoral and peritumoral PET/CT-based radiomics for non-invasively and dynamically predicting immunotherapy response in NSCLC
Source: Br J Cancer. 2025 Feb 10;132(6):558–68. doi: 10.1038/s41416-025-02948-z (PMC11920075; doi:10.1038/s41416-025-02948-z)
Supplement: Supplementary file 4 — Supplementary Tables [file 41416_2025_2948_MOESM4_ESM.docx]

**Supplementary Tables**

**Table S1.** The radiomic features and parameters of four radiomic models

| **The radiomic features of four radiomic models（Sort descending according to the importance of the radiomic features）** | | | | | | | |
| --- | --- | --- | --- | --- | --- | --- | --- |
| PET-Radscore  (15 features) | | CT-Radsocre  (11 features) | | PET/CT-Radsocre  (7 features) | | COMB-Radsocre  (25 features) | |
| PET Peritumor square glcm JointEnergy | | CT Intratumor lbp 3D k firstorder Variance | | PET/CT Intratumor wavelet LLH firstorder Mean | | PET/CT Intratumor wavelet LLH firstorder Mean | |
| PET TumorPeritumor log sigma 5.0 mm 3D glszm GrayLevelNonUniformityNormalized | | CT Intratumor log sigma 1.0 mm 3D gldm DependenceVariance | | PET/CT Peritumor wavelet LLL glcm Idmn | | PET TumorPeritumor log sigma 4.0 mm 3D glszm GrayLevelNonUniformityNormalized | |
| PET TumorPeritumor square glszm LowGrayLevelZoneEmphasis | | CT Peritumor gradient firstorder 10Percentile | | PET/CT Intratumor original glszm GrayLevelNonUniformity | | CT Intratumor lbp 3D k firstorder Variance | |
| PET Intratumor wavelet HHH firstorder InterquartileRange | | CT Intratumor original ngtdm Busyness | | PET/CT TumorPeritumor wavelet HHH gldm SmallDependenceLowGrayLevelEmphasis | | PET Tumor wavelet LLH gldm HighGrayLevelEmphasis | |
| PET TumorPeritumor log sigma 5.0 mm 3D glszm LowGrayLevelZoneEmphasis | | CT Intratumor wavelet HLH glcm SumSquares | | PET/CT Intratumor wavelet HLH firstorder Variance | | PET/CT Tumor wavelet HHL glcm MCC | |
| PET Tumor wavelet LHH glcm ClusterShade | | CT Peritumor wavelet HHH firstorder Maximum | | PET/CT Tumor wavelet HHH gldm SmallDependenceLowGrayLevelEmphasis | | CT Intratumor log sigma 1.0 mm 3D gldm DependenceVariance | |
| PET TumorPeritumor wavelet LHL glrlm LowGrayLevelRunEmphasis | | CT Tumor wavelet HHH glrlm LongRunHighGrayLevelEmphasis | | PET/CT Intratumor square glszm SmallAreaEmphasis | | PET/CT TumorPeritumor wavelet HHH glszm HighGrayLevelZoneEmphasis | |
| PET Tumor wavelet HLL glszm LargeAreaLowGrayLevelEmphasis | | CT Intratumor lbp 3D k firstorder Skewness | |  | | PET/CT Intratumor original glszm GrayLevelNonUniformity | |
| PET Peritumor gradient firstorder Minimum | | CT Peritumor square glszm SizeZoneNonUniformityNormalized | |  | | PET Intratumor wavelet HHH firstorder InterquartileRange | |
| PET Intratumor wavelet LLH glcm ClusterShade | | CT Intratumor wavelet HLH firstorder 90Percentile | |  | | PET/CT TumorPeritumor wavelet LLL gldm DependenceVariance | |
| PET Peritumor log sigma 5.0 mm 3D glrlm ShortRunEmphasis | | CT Tumor wavelet HLL firstorder Mean | |  | | PET/CT Intratumor wavelet HLH firstorder 10Percentile | |
| PET TumorPeritumor log sigma 4 0 mm 3D glszm GrayLevelNonUniformityNormalized | |  | |  | | PET Intratumor wavelet LLH glcm ClusterShade | |
| PET Tumor log sigma 5.0 mm 3D glszm LowGrayLevelZoneEmphasis | |  | |  | | PET Tumor log sigma 5.0 mm 3D glszm LowGrayLevelZoneEmphasis | |
| PET Tumor wavelet LLH gldm HighGrayLevelEmphasis | |  | |  | | PET/CT Intratumor square glszm SizeZoneNonUniformityNormalized | |
| PET Tumor lbp 3D m1 glrlm ShortRunLowGrayLevelEmphasis | |  | |  | | PET TumorPeritumor wavelet HLH firstorder Skewness | |
|  | |  | |  | | PET Tumor lbp 3D m1 glrlm ShortRunLowGrayLevelEmphasis | |
|  | |  | |  | | PET Peritumor wavelet LLH glcm ClusterShade | |
|  | |  | |  | | PET Tumor wavelet LHH glcm ClusterShade | |
|  | |  | |  | | PET Tumor wavelet HHL gldm LargeDependenceLowGrayLevelEmphasis | |
|  | |  | |  | | PET/CT Peritumor exponential ngtdm Strength | |
|  | |  | |  | | PET/CT Intratumor original gldm SmallDependenceHighGrayLevelEmphasis | |
|  | |  | |  | | PET TumorPeritumor log sigma 5.0 mm 3D glszm GrayLevelNonUniformityNormalized | |
|  | |  | |  | | PET TumorPeritumor log sigma 1.0 mm 3D glszm SmallAreaLowGrayLevelEmphasis | |
|  | |  | |  | | PET/CT Peritumor wavelet HLH glrlm LongRunHighGrayLevelEmphasis | |
|  | |  | |  | | PET Peritumor log sigma 5.0 mm 3D glrlm ShortRunEmphasis | |
| **The parameters of four radiomic models（The remaining parameters not listed have default values）** | | | | | | | |
| Parameters | PET-Radscore | | CT-Radsocre | | PET/CT-Radsocre | | COMB-Radsocre |
| nround | 6 | | 8 | | 5 | | 10 |
| max_depth | 3 | | 3 | | 3 | | 3 |
| eta | 0.400 | | 0.350 | | 0.375 | | 0.300 |
| subsample | 0.3 | | 0.3 | | 0.3 | | 0.3 |

**Table S2.** Demographic and clinicopathological characteristics of patients

|  | **Training cohort (N=183)** | | | ***p*^1^** | **Testing cohort (N=78)** | | | ***p*^1^** | ***p*^2^** | **All patients**  **(%)** | ***p*^3^** |
| --- | --- | --- | --- | --- | --- | --- | --- | --- | --- | --- | --- |
|  | **DCB** | **NDB** | **Total (%)** |  | **DCB** | **NDB** | **Total (%)** |  |  | **(n=261)** |  |
|  | **(n=126)** | **(n=57)** | **(n=183)** |  | **(n=58)** | **(n=20)** | **(n=78)** |  |  |  |  |
| **Sex** |  |  |  | **0.699** |  |  |  | **1.000** | **0.644** |  | **0.375** |
| Male | 109 (86.51%) | 46 (80.70%) | 155 (84.70%) |  | 48 (82.76%) | 14 (70.00%) | 62 (79.49%) |  |  | 217（83.14） |  |
| Female | 17 (13.49%) | 11 (19.30%) | 28 (15.30%) |  | 10 (17.24%) | 6 (30.00%) | 16 (20.51%) |  |  | 44 (16.86%) |  |
| **Age** |  |  |  | **0.851** |  |  |  | **0.556** | **0.749** |  | **0.806** |
| ≤55 | 31 (24.60%) | 13 (22.81%) | 44 (24.04%) |  | 9 (15.52%) | 7 (35.00%) | 16 (20.51%) |  |  | 60 (22.99%) |  |
| 56–65 | 45 (35.71%) | 24 (42.11%) | 69 (37.70%) |  | 21 (36.21%) | 5 (25.00%) | 26 (33.33%) |  |  | 95 (36.40%) |  |
| >65 | 50 (39.68%) | 20 (35.09%) | 70 (38.25%) |  | 28 (48.28%) | 8 (40.00%) | 36 (46.15%) |  |  | 106 (40.61%) |  |
| **BMI** |  |  |  | **0.068** |  |  |  | **0.472** | **0.894** |  | **0.022** |
| Mean(SD) | 22.53 (3.17) | 21.42 (2.55) | 22.19 (3.03) |  | 22.36 (2.99) | 21.09 (2.49) | 22.04 (2.90) |  |  | 22.14  (2.99) |  |
| **Smoke** |  |  |  | **0.851** |  |  |  | **1.000** | **0.644** |  | **0.861** |
| Non | 27 (21.43%) | 9 (15.79%) | 36 (19.67%) |  | 16 (27.59%) | 7 (35.00%) | 23 (29.49%) |  |  | 59 (22.61%) |  |
| Former | 23 (18.25%) | 10 (17.54%) | 33 (18.03%) |  | 12 (20.69%) | 3 (15.00%) | 15 (19.23%) |  |  | 48 (18.39%) |  |
| Current | 76 (60.32%) | 38 (66.67%) | 114 (62.30%) |  | 30 (51.72%) | 10 (50.00%) | 40 (51.28%) |  |  | 154 (59.00%) |  |
| **Histology** |  |  |  | **0.071** |  |  |  | **1.000** | **0.494** |  | **0.475** |
| ADC | 71 (56.35%) | 23 (40.35%) | 94 (51.37%) |  | 37 (63.79%) | 15 (75.00%) | 52 (66.67%) |  |  | 146 (55.94%) |  |
| SCC | 48 (38.10%) | 25 (43.86%) | 73 (39.89%) |  | 13 (22.41%) | 4 (20.00%) | 17 (21.79%) |  |  | 90 (34.48%) |  |
| Other | 7 (5.56%) | 9 (15.79%) | 16 (8.74%) |  | 8 (13.79%) | 1  (5.00%) | 9 (11.54%) |  |  | 25  (9.58%) |  |
| **T stage** |  |  |  | **0.851** |  |  |  | **0.295** | **0.644** |  | **0.216** |
| 1–3 | 65 (51.59%) | 27 (47.37%) | 92 (50.27%) |  | 38 (65.52%) | 7 (35.00%) | 45 (57.69%) |  |  | 137（52.49%） |  |
| 4 | 61 (48.41%) | 30 (52.63%) | 91 (49.73%) |  | 20 (34.48%) | 13 (65.00%) | 33 (42.31%) |  |  | 124 (47.51%) |  |
| **N stage** |  |  |  | **0.949** |  |  |  | **1.000** | **1.000** |  | **0.861** |
| 0–2 | 42 (33.33%) | 18 (31.58%) | 60 (32.79%) |  | 21 (36.21%) | 6 (30.00%) | 27 (34.62%) |  |  | 87 (33.33%) |  |
| 3 | 84 (66.67%) | 39 (68.42%) | 123 (67.21%) |  | 37 (63.79%) | 14 (70.00%) | 51 (65.38%) |  |  | 174 (66.67%) |  |
| **M stage** |  |  |  | **0.851** |  |  |  | **1.000** | **0.644** |  | **0.974** |
| M0 | 31 (24.60%) | 16 (28.07%) | 47 (25.68%) |  | 19 (32.76%) | 6 (30.00%) | 25 (32.05%) |  |  | 72 (27.59%) |  |
| M1 | 95 (75.40%) | 41 (71.93%) | 136 (74.32%) |  | 39 (67.24%) | 14 (70.00%) | 53 (67.95%) |  |  | 189 (72.41%) |  |
| **Stage** |  |  |  | **0.841** |  |  |  | **1.000** | **0.644** |  | **0.861** |
| III | 29 (23.02%) | 16 (28.07%) | 45 (24.59%) |  | 19 (32.76%) | 6 (30.00%) | 25 (32.05%) |  |  | 70 (26.82%) |  |
| IV | 97 (76.98%) | 41 (71.93%) | 138 (75.41%) |  | 39 (67.24%) | 14 (70.00%) | 53 (67.95%) |  |  | 191 (73.18%) |  |
| **Metastases** |  |  |  | **0.078** |  |  |  | **1.000** | **0.947** |  | **0.210** |
| <3 | 94 (74.60%) | 51 (89.47%) | 145 (79.23%) |  | 45 (77.59%) | 15 (75.00%) | 60 (76.92%) |  |  | 205 (78.54%) |  |
| ≥3 | 32 (25.40%) | 6 (10.53%) | 38 (20.77%) |  | 13 (22.41%) | 5 (25.00%) | 18 (23.08%) |  |  | 56 (21.46%) |  |
| **Treatment strategy** |  |  |  | **0.071** |  |  |  | **1.000** | **1.000** |  | **0.164** |
| Combination therapy | 117 (92.86%) | 46 (80.70%) | 163 (89.07%) |  | 52 (89.66%) | 18 (90.00%) | 70 (89.74%) |  |  | 233 (89.27%) |  |
| Monotherapy | 9 (7.14%) | 11 (19.30%) | 20 (10.93%) |  | 6 (10.34%) | 2 (10.00%) | 8 (10.26%) |  |  | 28 (10.73%) |  |
| **Local radiotherapy** |  |  |  | **0.757** |  |  |  | **0.472** | **0.644** |  | **0.175** |
| No | 114 (90.48%) | 54 (94.74%) | 168 (91.80%) |  | 48 (82.76%) | 20 (100.00%) | 68 (87.18%) |  |  | 236 (90.42%) |  |
| Yes | 12 (9.52%) | 3 (5.26%) | 15 (8.20%) |  | 10 (17.24%) | 0  (0.00%) | 10 (12.82%) |  |  | 25  (9.58%) |  |
| **irAE** |  |  |  | **0.586** |  |  |  | **1.000** | **1.000** |  | **0.609** |
| No | 104 (82.54%) | 51 (89.47%) | 155 (84.70%) |  | 50 (86.21%) | 17 (85.00%) | 67 (85.90%) |  |  | 222 (85.06%) |  |
| Yes | 22 (17.46%) | 6 (10.53%) | 28 (15.30%) |  | 8 (13.79%) | 3  (15.00%) | 11 (14.10%) |  |  | 39 (14.94%) |  |
| **PD-L1(TPS)** |  |  |  | **0.042** |  |  |  | **0.273** | **0.644** |  | **0.013** |
| Median[IQR] | 20.00  [3.00, 57.50] | 1.00  [1.00, 30.00] | 15.00  [1.00, 50.00] |  | 20.00  [1.00, 33.75] | 1.00  [0.00, 11.25] | 17.50  [0.25, 28.75] |  |  | 15.00  [1.00, 40.00] |  |
| **CEA** |  |  |  | **0.851** |  |  |  | **1.000** | **0.644** |  | **0.985** |
| Median[IQR] | 4.91  [2.70, 15.94] | 5.17  [2.29, 13.01] | 4.91  [2.60, 15.46] |  | 5.47  [2.67, 32.55] | 7.82  [4.68, 14.17] | 6.74  [2.80, 29.10] |  |  | 5.01  [2.65, 19.11] |  |
| **CYFRA211** |  |  |  | **0.013** |  |  |  | **1.000** | **0.644** |  | **0.013** |
| Median[IQR] | 5.30  [2.83, 9.36] | 9.19  [5.03, 31.83] | 5.89  [3.38, 13.38] |  | 5.30  [4.02, 15.17] | 9.13  [6.29, 12.93] | 7.07  [4.02, 15.17] |  |  | 6.03  [3.64, 13.60] |  |
| **NSE** |  |  |  | **0.061** |  |  |  | **0.553** | **0.644** |  | **0.022** |
| Median[IQR] | 10.83  [5.58, 14.58] | 13.58  [7.65, 18.20] | 10.83  [6.00, 16.23] |  | 11.04  [4.97, 17.52] | 14.25  [10.79, 17.54] | 12.07  [6.00, 17.76] |  |  | 11.23  [5.94, 16.72] |  |
| **SCCA** |  |  |  | **0.013** |  |  |  | **1.000** | **1.000** |  | **0.013** |
| Median[IQR] | 0.88  [0.31, 2.15] | 1.36  [0.80, 4.37] | 1.01  [0.41, 2.67] |  | 0.96  [0.41, 2.29] | 0.84  [0.50, 1.95] | 0.90  [0.41, 2.29] |  |  | 0.98  [0.41, 2.38] |  |
| **WBC** |  |  |  | **0.586** |  |  |  | **1.000** | **0.771** |  | **0.609** |
| Median[IQR] | 8.49  [6.54, 10.29] | 8.63  [7.30, 10.23] | 8.57  [6.74, 10.29] |  | 8.08  [7.15, 9.85] | 7.91  [7.02, 10.31] | 8.05  [7.07, 10.24] |  |  | 8.44  [6.77, 10.27] |  |
| **MNC** |  |  |  | **0.943** |  |  |  | **1.000** | **0.644** |  | **0.861** |
| Median[IQR] | 0.63 [0.46, 0.84] | 0.59 [0.47, 0.90] | 0.62 [0.46, 0.84] |  | 0.65  [0.52, 0.92] | 0.67  [0.55, 0.79] | 0.66  [0.52, 0.87] |  |  | 0.63  [0.48, 0.85] |  |
| **LYM** |  |  |  | **0.948** |  |  |  | **0.423** | **0.644** |  | **0.557** |
| Median[IQR] | 1.60  [1.22, 2.07] | 1.59  [1.29, 1.99] | 1.60  [1.23, 2.04] |  | 1.56  [1.20, 1.92] | 1.35  [1.12, 1.59] | 1.49  [1.17, 1.84] |  |  | 1.57  [1.22, 1.98] |  |
| **NEU** |  |  |  | **0.542** |  |  |  | **1.000** | **0.894** |  | **0.475** |
| Median[IQR] | 5.68  [4.08, 7.50] | 6.13  [4.48, 7.71] | 5.76  [4.20, 7.68] |  | 5.76  [4.27, 7.63] | 5.35  [5.02, 7.94] | 5.61  [4.35, 7.64] |  |  | 5.73  [4.27, 7.65] |  |
| **PLT** |  |  |  | **0.042** |  |  |  | **1.000** | **0.644** |  | **0.124** |
| Median[IQR] | 275.50  [220.50, 339.50] | 308.00  [250.00, 370.00] | 291.00  [233.00, 356.50] |  | 284.50 [244.50, 400.00] | 287.50  [245.75, 377.25] | 287.50 [244.50, 379.75] |  |  | 290.00  [235.00, 360.00] |  |
| **ALB** |  |  |  | **0.071** |  |  |  | **0.273** | **0.644** |  | **0.013** |
| Mean(SD) | 39.22 (4.70) | 37.57 (4.51) | 38.71 (4.69) |  | 38.77 (5.30) | 35.63 (3.85) | 37.96 (5.13) |  |  | 38.48  (4.83) |  |
| **CRP** |  |  |  | **0.042** |  |  |  | **1.000** | **0.644** |  | **0.048** |
| Median[IQR] | 8.56  [3.41, 30.32] | 27.83  [6.63, 51.70] | 12.49  [3.72, 35.95] |  | 14.44  [3.50, 56.87] | 14.92  [7.12, 44.78] | 14.44  [4.59, 54.39] |  |  | 13.77  [3.86, 38.97] |  |
| **LDH** |  |  |  | **0.063** |  |  |  | **1.000** | **0.894** |  | **0.098** |
| Median[IQR] | 186.00  [160.50, 212.75] | 207.50  [165.00, 255.00] | 187.00 [162.00, 223.50] |  | 191.00  [164.25, 236.00] | 203.75  [175.00, 221.50] | 195.00  [165.00, 226.00] |  |  | 188.00  [163.00, 224.00] |  |

DCB, durable clinical benefit; NDB, no durable clinical benefit; BMI, body mass index; Non, never smoked before; Former, quit smoking for more than 3 months before treatment; Current, still smoking for more than 3 months before treatment; ADC, adenocarcinoma; SCC, squamous cell carcinoma; Other, other pathological types except for adenocarcinoma and squamous cell carcinoma; T, tumor; N, node; M, metastasis; Combination therapy, combination therapy with immune checkpoint inhibitors, i.e., PD1 (e.g., pembrolizumab or nivolumab)/PD-L1 (e.g., durvalumab) ICI in combination with chemotherapeutic agents (e.g., pemetrexed/paclitaxel/gemcitabine+cisplatin/carboplatin/lobaplatin) and/or antiangiogenic agents (e.g., bevacizumab); Monotherapy, immune checkpoint inhibitor monotherapy, i.e., PD1 (e.g., pembrolizumab or nivolumab)/PD-L1 (e.g., durvalumab) ICI monotherapy; irAE, immune-related adverse event; PD-L1 (TPS), programmed death-ligand 1 (tumor proportion score); CEA, carcinoembryonic antigen; CYFRA211, cytokeratin 19 fragment antigen 211; NSE, neuron-specific enolase; SCCA, squamous cell carcinoma antigen; WBC, white blood cells; MNC, mononuclear cells; LYM, lymphocyte; NEU, neutrophil; PLT, platelet; ALB, albumin; CRP, C-reactive protein; LDH, lactate dehydrogenase.

Categorical variable values are presented as numbers (%). Continuous variable values satisfying normal distribution are presented as mean (standard deviation); others are presented as median [interquartile range].

*p*^1^ derived from the *t* test (continuous variable) and chi-square test or Fisher's exact test (categorical variable) between DCB and NDB of the training and testing cohorts, respectively.

*p*^2^ derived from the *t* test (continuous variable) and chi-square test or Fisher's exact test (categorical variable) between training and testing cohorts.

*p*^3^ derived from the *t* test (continuous variable) and chi-square test or Fisher's exact test (categorical variable) between DCB and NDB of the total study population.

The p-values were subjected to multiple hypothesis testing corrections utilizing the Benjamini-Hochberg method.

*p* < 0.05 was considered to indicate a significant statistical difference.

**Table S3.** NRI and IDI analysis of four radiomic models

|  | **Training cohort（N=183）** | | | |  | **Testing cohort（N=78）** | | | |
| --- | --- | --- | --- | --- | --- | --- | --- | --- | --- |
|  | **NRI (95%CI)** | ***p*** | **IDI (95% CI)** | ***p*** |  | **NRI (95% CI)** | ***p*** | **IDI (95% CI)** | ***p*** |
| COMB vs PET(Reference) | 0.09  （-0.09，0.26） | 0.335 | 0.04  （-0.03，0.12） | 0.260 |  | 0.34  （0.02，0.65） | 0.035 | 0.21  （0.06，0.36） | 0.007 |
| COMB vs CT(Reference) | 0.10  （-0.06，0.26） | 0.210 | 0.07  （0.00，0.14） | 0.060 |  | 0.29  （0.02，0.56） | 0.038 | 0.14  （0.03，0.25） | 0.012 |
| COMB vs PET/CT(Reference) | 0.21  （0.06，0.35） | 0.004 | 0.13  （0.07，0.19） | <0.001 |  | 0.25  （-0.06，0.57） | 0.111 | 0.17  （0.05，0.28） | 0.006 |

CI, confidence interval; NRI, net reclassification improvement; IDI, integrated discrimination improvement.

**Table S4.** Positive Predictive Value (PPV), Negative Predictive Value (NPV), Sensitivity, Specificity, Accuracy, Recall, F1 Score, Matthews Correlation Coefficient (MCC), and Kappa for four radiomic models

|  | **Training cohort（N=183）** | | | |  | **Testing cohort（N=78）** | | | |
| --- | --- | --- | --- | --- | --- | --- | --- | --- | --- |
|  | **PET** | **CT** | **PET/CT** | **COMB** |  | **PET** | **CT** | **PET/CT** | **COMB** |
| Cut-off Value | -0.470 | -0.678 | -0.579 | -0.617 |  | -0.470 | -0.678 | -0.579 | -0.617 |
| TP | 43 | 48 | 41 | 47 |  | 9 | 10 | 11 | 14 |
| TN | 104 | 92 | 93 | 106 |  | 42 | 43 | 41 | 47 |
| FP | 22 | 34 | 33 | 20 |  | 16 | 15 | 17 | 11 |
| FN | 14 | 9 | 16 | 10 |  | 11 | 10 | 9 | 6 |
| PPV | 0.66 | 0.59 | 0.55 | 0.70 |  | 0.36 | 0.40 | 0.39 | 0.56 |
| NPV | 0.88 | 0.91 | 0.85 | 0.91 |  | 0.79 | 0.81 | 0.82 | 0.89 |
| Sensitivity | 0.75 | 0.84 | 0.72 | 0.82 |  | 0.45 | 0.50 | 0.55 | 0.70 |
| Specificity | 0.83 | 0.73 | 0.74 | 0.84 |  | 0.72 | 0.74 | 0.71 | 0.81 |
| Accuracy | 0.80 | 0.77 | 0.73 | 0.84 |  | 0.65 | 0.68 | 0.67 | 0.78 |
| Precision | 0.66 | 0.59 | 0.55 | 0.70 |  | 0.36 | 0.40 | 0.39 | 0.56 |
| Recall | 0.75 | 0.84 | 0.72 | 0.82 |  | 0.45 | 0.50 | 0.55 | 0.70 |
| F1 score | 0.70 | 0.69 | 0.63 | 0.76 |  | 0.40 | 0.44 | 0.46 | 0.62 |
| MCC | 0.56 | 0.53 | 0.43 | 0.64 |  | 0.16 | 0.23 | 0.23 | 0.48 |
| Kappa | 0.56 | 0.51 | 0.42 | 0.64 |  | 0.16 | 0.22 | 0.23 | 0.47 |

TP, true positives；TN, true negatives；FP, false positives；FN, false negatives；PPV, positive predictive value; NPV, negative predictive value; MCC, matthews correlation coefficient.

**Table S5.** Univariate and multivariate cox regression of COMB-Radscore, clinicopathological characteristics with progression-free survival in the training cohort

| **Characteristic** | **Univariate analysis** | |  | **Multivariate analysis** | |
| --- | --- | --- | --- | --- | --- |
|  | **HR（95%CI）** | ***p*** |  | **HR（95%CI）** | ***p*** |
| COMB-Radscore | 1.78 (1.51-2.1) | <0.001 |  | 1.8 (1.51-2.14) | <0.001 |
| Sex |  |  |  |  |  |
| Male | Reference | — |  | — | — |
| Female | 1.25 (0.75-2.1) | 0.394 |  | — | — |
| Age |  |  |  |  |  |
| ≤55 | Reference | — |  | — | — |
| 56–65 | 1.35 (0.81-2.24) | 0.243 |  |  |  |
| >65 | 1.09 (0.64-1.83) | 0.755 |  |  |  |
| BMI | 0.93 (0.88-1) | 0.043 |  | 0.98 (0.91-1.04) | 0.456 |
| Smoke |  |  |  |  |  |
| Non | Reference | — |  | — | — |
| Former | 1.24 (0.65-2.33) | 0.514 |  | — | — |
| Current | 1.19 (0.71-1.97) | 0.511 |  | — | — |
| Histology |  |  |  |  |  |
| ADC | Reference | — |  | — | — |
| SCC | 1.3 (0.86-1.97) | 0.208 |  | 1.14 (0.74-1.75) | 0.550 |
| Other | 2.44 (1.23-4.83) | 0.01 |  | 2.76 (1.26-6.06) | 0.011 |
| T stage |  |  |  |  |  |
| 1–3 | Reference | — |  | — | — |
| 4 | 1.14 (0.77-1.67) | 0.521 |  | — | — |
| N stage |  |  |  |  |  |
| 0–2 | Reference | — |  | — | — |
| 3 | 1.08 (0.71-1.65) | 0.704 |  | — | — |
| M stage |  |  |  |  |  |
| M0 | Reference | — |  | — | — |
| M1a+M1b | 0.97 (0.57-1.66) | 0.908 |  | — | — |
| M1c | 1.14 (0.7-1.86) | 0.595 |  | — | — |
| Stage |  |  |  |  |  |
| III | Reference | — |  | — | — |
| IV | 1.07 (0.68-1.69) | 0.774 |  | — | — |
| Metastases |  |  |  |  |  |
| <3 | Reference | — |  | — | — |
| ≥3 | 0.77 (0.46-1.28) | 0.314 |  | — | — |
| Treatment strategy |  |  |  |  |  |
| Monotherapy | Reference | — |  | — | — |
| Combination therapy | 0.73 (0.41-1.31) | 0.295 |  | — | — |
| Local radiotherapy |  |  |  |  |  |
| No | Reference | — |  | — | — |
| Yes | 0.82 (0.4-1.7) | 0.6 |  | — | — |
| irAE |  |  |  |  |  |
| No | Reference | — |  | — | — |
| Yes | 0.66 (0.37-1.16) | 0.148 |  | — | — |
| PD-L1(TPS) | 0.99 (0.98-1) | 0.004 |  | 0.99 (0.98-1) | 0.004 |
| CEA | 1 (1-1) | 0.139 |  | — | — |
| CYFRA211 | 1.01 (1.01-1.02) | <0.001 |  | 1.01 (1-1.01) | 0.002 |
| NSE | 1.02 (1.01-1.03) | <0.001 |  | 1.01 (1-1.02) | 0.171 |
| SCCA | 1.01 (0.99-1.02) | 0.434 |  | — | — |
| WBC | 1.03 (0.96-1.09) | 0.432 |  | — | — |
| MNC | 0.89 (0.48-1.65) | 0.716 |  | — | — |
| LYM | 0.82 (0.59-1.13) | 0.216 |  | — | — |
| NEU | 1.04 (0.97-1.11) | 0.269 |  | — | — |
| PLT | 1 (1-1) | 0.076 |  | — | — |
| ALB | 0.95 (0.91-0.99) | 0.01 |  | 0.95 (0.91-0.99) | 0.027 |
| CRP | 1 (1-1.01) | 0.193 |  | — | — |
| LDH | 1 (1-1) | 0.009 |  | 1 (1-1) | 0.517 |

BMI, body mass index; Non, never smoked before; Former, quit smoking for more than 3 months before treatment; Current, still smoking for more than 3 months before treatment; ADC, adenocarcinoma; SCC, squamous cell carcinoma; Other, other pathological types except for adenocarcinoma and squamous cell carcinoma; T, tumor; N, node; M, metastasis; Combination therapy, combination therapy with immune checkpoint inhibitors, i.e., PD1 (e.g., pembrolizumab or nivolumab)/PD-L1 (e.g., durvalumab) ICI in combination with chemotherapeutic agents (e.g., pemetrexed/paclitaxel/gemcitabine+cisplatin/carboplatin/lobaplatin) and/or antiangiogenic agents (e.g., bevacizumab); Monotherapy, immune checkpoint inhibitor monotherapy, i.e., PD1 (e.g., pembrolizumab or nivolumab)/PD-L1 (e.g., durvalumab) ICI monotherapy; irAE, immune-related adverse event; PD-L1(TPS), programmed death-ligand 1(tumor proportion score); CEA, carcinoembryonic antigen; CYFRA211, Cytokeratin 19 fragment antigen 211; NSE, neuron-specific enolase; SCCA, squamous cell carcinoma antigen; WBC, white blood cell; MNC, mononuclear cells; LYM, lymphocyte; NEU, neutrophil; PLT, platelet; ALB, albumin; CRP, c-reactive protein; LDH, lactate dehydrogenase; HR, hazard ratio; CI, confidence interval.

**Table S6.** Univariate and multivariate cox regression of COMB-Radscore, clinicopathological characteristics with progression-free survival in the testing cohort

| **Characteristic** | **Univariate analysis** | |  | **Multivariate analysis** | |
| --- | --- | --- | --- | --- | --- |
|  | **HR（95%CI）** | ***p*** |  | **HR（95%CI）** | ***p*** |
| COMB-Radscore | 1.7 (1.29-2.24) | <0.001 |  | 2 (1.46-2.75) | <0.001 |
| Sex |  |  |  |  |  |
| Male | Reference | — |  | — | — |
| Female | 1.78 (0.88-3.61) | 0.110 |  | — | — |
| Age |  |  |  |  |  |
| ≤55 | Reference | — |  | — | — |
| 56–65 | 0.34 (0.14-0.86) | 0.022 |  | 0.25 (0.09-0.67) | 0.006 |
| >65 | 0.63 (0.29-1.34) | 0.230 |  | 0.55 (0.25-1.21) | 0.140 |
| BMI | 0.96 (0.87-1.06) | 0.382 |  | — | — |
| Smoke |  |  |  |  |  |
| Non | Reference | — |  | — | — |
| Former | 0.55 (0.18-1.73) | 0.309 |  | — | — |
| Current | 1.28 (0.64-2.55) | 0.483 |  | — | — |
| Histology |  |  |  |  |  |
| ADC | Reference | — |  | — | — |
| SCC | 0.74 (0.34-1.57) | 0.426 |  | — | — |
| Other | 0.38 (0.11-1.28) | 0.119 |  | — | — |
| T stage |  |  |  |  |  |
| 1–3 | Reference | — |  | — | — |
| 4 | 1.94 (1.05-3.6) | 0.035 |  | 1.88 (0.97-3.63) | 0.061 |
| N stage |  |  |  |  |  |
| 0–2 | Reference | — |  | — | — |
| 3 | 0.97 (0.51-1.84) | 0.925 |  | — | — |
| M stage |  |  |  |  |  |
| M0 | Reference | — |  | — | — |
| M1a+M1b | 1.1 (0.51-2.37) | 0.809 |  | — | — |
| M1c | 1.04 (0.47-2.29) | 0.924 |  | — | — |
| Stage |  |  |  |  |  |
| III | Reference | — |  | — | — |
| IV | 1.07 (0.54-2.12) | 0.846 |  | — | — |
| Metastases |  |  |  |  |  |
| <3 | Reference | — |  | — | — |
| ≥3 | 0.86 (0.39-1.87) | 0.699 |  | — | — |
| Treatment strategy |  |  |  |  |  |
| Monotherapy | Reference | — |  | — | — |
| Combination therapy | 1.22 (0.43-3.47) | 0.705 |  | — | — |
| Local radiotherapy |  |  |  |  |  |
| No | Reference | — |  | — | — |
| Yes | 0.86 (0.37-2) | 0.730 |  | — | — |
| irAE |  |  |  |  |  |
| No | Reference | — |  | — | — |
| Yes | 1.47 (0.64-3.34) | 0.362 |  | — | — |
| PD-L1(TPS) | 0.99 (0.98-1.01) | 0.308 |  | — | — |
| CEA | 1 (1-1) | 0.408 |  | — | — |
| CYFRA211 | 0.99 (0.98-1) | 0.197 |  | — | — |
| NSE | 1.01 (0.99-1.02) | 0.492 |  | — | — |
| SCCA | 1.02 (0.99-1.05) | 0.208 |  | — | — |
| WBC | 0.94 (0.85-1.04) | 0.247 |  | — | — |
| MNC | 0.62 (0.22-1.76) | 0.369 |  | — | — |
| LYM | 0.65 (0.35-1.21) | 0.177 |  | — | — |
| NEU | 0.96 (0.86-1.06) | 0.401 |  | — | — |
| PLT | 1 (1-1) | 0.673 |  | — | — |
| ALB | 0.95 (0.89-1.01) | 0.078 |  | — | — |
| CRP | 1 (0.99-1.01) | 0.980 |  | — | — |
| LDH | 1 (1-1) | 0.286 |  | — | — |

BMI, body mass index; Non, never smoked before; Former, quit smoking for more than 3 months before treatment; Current, still smoking for more than 3 months before treatment; ADC, adenocarcinoma; SCC, squamous cell carcinoma; Other, other pathological types except for adenocarcinoma and squamous cell carcinoma; T, tumor; N, node; M, metastasis; Combination therapy, combination therapy with immune checkpoint inhibitors, i.e., PD1 (e.g., pembrolizumab or nivolumab)/PD-L1 (e.g., durvalumab) ICI in combination with chemotherapeutic agents (e.g., pemetrexed/paclitaxel/gemcitabine+cisplatin/carboplatin/lobaplatin) and/or antiangiogenic agents (e.g., bevacizumab); Monotherapy, immune checkpoint inhibitor monotherapy, i.e., PD1 (e.g., pembrolizumab or nivolumab)/PD-L1 (e.g., durvalumab) ICI monotherapy; irAE, immune-related adverse event; PD-L1(TPS), programmed death-ligand 1(tumor proportion score); CEA, carcinoembryonic antigen; CYFRA211, cytokeratin 19 fragment antigen 211; NSE, neuron-specific enolase; SCCA, squamous cell carcinoma antigen; WBC, white blood cell; MNC, mononuclear cells; LYM, lymphocyte; NEU, neutrophil; PLT, platelet; ALB, albumin; CRP, c-reactive protein; LDH, lactate dehydrogenase; HR, hazard ratio; CI, confidence interval.

**Table S7.** Univariate and multivariate cox regression of COMB-Radscore, clinicopathological characteristics with overall survival in the training cohort

| **Characteristic** | **Univariate analysis** | |  | **Multivariate analysis** | |
| --- | --- | --- | --- | --- | --- |
|  | **HR（95%CI）** | ***p*** |  | **HR（95%CI）** | ***p*** |
| COMB-Radscore | 1.71 (1.29-2.28) | <0.001 |  | 1.7 (1.24-2.32) | <0.001 |
| Sex |  |  |  |  |  |
| Male | Reference | — |  | — | — |
| Female | 0.34 (0.08-1.43) | 0.141 |  | — | — |
| Age |  |  |  |  |  |
| ≤55 | Reference | — |  | — | — |
| 56–65 | 1.52 (0.58-3.97) | 0.390 |  | — | — |
| >65 | 2.16 (0.83-5.59) | 0.114 |  | — | — |
| BMI | 0.95 (0.85-1.06) | 0.328 |  | — | — |
| Smoke |  |  |  |  |  |
| Non | Reference | — |  | — | — |
| Former | 3.06 (0.81-11.57) | 0.100 |  | — | — |
| Current | 2.95 (0.88-9.92) | 0.080 |  | — | — |
| Histology |  |  |  |  |  |
| ADC | Reference | — |  | — | — |
| SCC | 0.66 (0.3-1.44) | 0.301 |  | 0.71 (0.28-1.77) | 0.458 |
| Other | 3.1 (1.03-9.27) | 0.043 |  | 3.27 (1.07-9.95) | 0.037 |
| T stage |  |  |  |  |  |
| 1–3 | Reference | — |  | — | — |
| 4 | 0.91 (0.47-1.77) | 0.782 |  | — | — |
| N stage |  |  |  |  |  |
| 0–2 | Reference | — |  | — | — |
| 3 | 1.24 (0.59-2.6) | 0.568 |  | — | — |
| M stage |  |  |  |  |  |
| M0 | Reference | — |  | — | — |
| M1a+M1b | 1.05 (0.31-3.62) | 0.938 |  | 0.83 (0.23-3.05) | 0.781 |
| M1c | 3.04 (1.05-8.81) | 0.041 |  | 2.79 (0.89-8.75) | 0.079 |
| Stage |  |  |  |  |  |
| III | Reference | — |  | — | — |
| IV | 2.14 (0.75-6.11) | 0.154 |  | — | — |
| Metastases |  |  |  |  |  |
| <3 | Reference | — |  | — | — |
| ≥3 | 1.05 (0.46-2.43) | 0.904 |  | — | — |
| Treatment strategy |  |  |  |  |  |
| Monotherapy | Reference | — |  | — | — |
| Combination therapy | 1.68 (0.51-5.5) | 0.393 |  | — | — |
| Local radiotherapy |  |  |  |  |  |
| No | Reference | — |  | — | — |
| Yes | 0.61 (0.15-2.55) | 0.496 |  | — | — |
| irAE |  |  |  |  |  |
| No | Reference | — |  | — | — |
| Yes | 0.77 (0.3-1.98) | 0.584 |  | — | — |
| PD-L1(TPS) | 0.98 (0.97-1) | 0.012 |  | 0.99 (0.97-1) | 0.039 |
| CEA | 1 (1-1) | 0.110 |  | — | — |
| CYFRA211 | 1.01 (1-1.02) | 0.003 |  | 1 (1-1.01) | 0.255 |
| NSE | 1 (0.98-1.03) | 0.744 |  | — | — |
| SCCA | 1.01 (0.99-1.03) | 0.311 |  | — | — |
| WBC | 1.05 (0.93-1.17) | 0.447 |  | — | — |
| MNC | 1.67 (0.68-4.14) | 0.266 |  | — | — |
| LYM | 0.61 (0.33-1.1) | 0.100 |  | — | — |
| NEU | 1.05 (0.94-1.19) | 0.380 |  | — | — |
| PLT | 1 (0.99-1) | 0.112 |  | — | — |
| ALB | 0.91 (0.85-0.98) | 0.013 |  | 0.9 (0.83-0.97) | 0.007 |
| CRP | 1 (1-1.01) | 0.362 |  | — | — |
| LDH | 1 (1-1) | 0.262 |  | — | — |

BMI, body mass index; Non, never smoked before; Former, quit smoking for more than 3 months before treatment; Current, still smoking for more than 3 months before treatment; ADC, adenocarcinoma; SCC, squamous cell carcinoma; Other, other pathological types except for adenocarcinoma and squamous cell carcinoma; T, tumor; N, node; M, metastasis; Combination therapy, combination therapy with immune checkpoint inhibitors, i.e., PD1 (e.g., pembrolizumab or nivolumab)/PD-L1 (e.g., durvalumab) ICI in combination with chemotherapeutic agents (e.g., pemetrexed/paclitaxel/gemcitabine+cisplatin/carboplatin/lobaplatin) and/or antiangiogenic agents (e.g., bevacizumab); Monotherapy, immune checkpoint inhibitor monotherapy, i.e., PD1 (e.g., pembrolizumab or nivolumab)/PD-L1 (e.g., durvalumab) ICI monotherapy; irAE, immune-related adverse event; PD-L1(TPS), programmed death-ligand 1(tumor proportion score); CEA, carcinoembryonic antigen; CYFRA211, cytokeratin 19 fragment antigen 211; NSE, neuron-specific enolase; SCCA, squamous cell carcinoma antigen; WBC, white blood cell; MNC, mononuclear cells; LYM, lymphocyte; NEU, neutrophil; PLT, platelet; ALB, albumin; CRP, c-reactive protein; LDH, lactate dehydrogenase; HR, hazard ratio; CI, confidence interval.

**Table S8.** Univariate and multivariate cox regression of COMB-Radscore, clinicopathological characteristics with overall survival in the testing cohort

| **Characteristic** | **Univariate analysis** | |  | **Multivariate analysis** | |
| --- | --- | --- | --- | --- | --- |
|  | **HR（95%CI）** | ***p*** |  | **HR（95%CI）** | ***p*** |
| COMB-Radscore | 1.98 (1.29-3.04) | 0.002 |  | 2.17 (1.31-3.58) | 0.003 |
| Sex |  |  |  |  |  |
| Male | Reference | — |  | — | — |
| Female | 0.93 (0.26-3.28) | 0.905 |  | — | — |
| Age |  |  |  |  |  |
| ≤55 | Reference | — |  | — | — |
| 56–65 | 0.67 (0.15-2.88) | 0.587 |  | — | — |
| >65 | 0.87 (0.23-3.26) | 0.836 |  | — | — |
| BMI | 0.89 (0.76-1.04) | 0.15 |  | — | — |
| Smoke |  |  |  |  |  |
| Non | Reference | — |  | — | — |
| Former | 0.85 (0.15-4.65) | 0.847 |  | — | — |
| Current | 1.66 (0.52-5.26) | 0.392 |  | — | — |
| Histology |  |  |  |  |  |
| ADC | Reference | — |  | — | — |
| SCC | 1.92 (0.65-5.64) | 0.235 |  | — | — |
| Other | 1.75 (0.46-6.64) | 0.413 |  | — | — |
| T stage |  |  |  |  |  |
| 1–3 | Reference | — |  | — | — |
| 4 | 2.95 (1.09-8.01) | 0.034 |  | 2.39 (0.75-7.63) | 0.142 |
| N stage |  |  |  |  |  |
| 0–2 | Reference | — |  | — | — |
| 3 | 1.29 (0.46-3.68) | 0.628 |  | — | — |
| M stage |  |  |  |  |  |
| M0 | Reference | — |  | — | — |
| M1a+M1b | 1.57 (0.46-5.41) | 0.473 |  | — | — |
| M1c | 1.75 (0.49-6.23) | 0.385 |  | — | — |
| Stage |  |  |  |  |  |
| III | Reference | — |  | — | — |
| IV | 1.65 (0.54-5.08) | 0.381 |  | — | — |
| Metastases |  |  |  |  |  |
| <3 | Reference | — |  | — | — |
| ≥3 | 1.35 (0.44-4.15) | 0.600 |  | — | — |
| Treatment strategy |  |  |  |  |  |
| Monotherapy | Reference | — |  | — | — |
| Combination therapy | 0.78 (0.22-2.72) | 0.692 |  | — | — |
| Local radiotherapy |  |  |  |  |  |
| No | Reference | — |  | — | — |
| Yes | 0.99 (0.28-3.55) | 0.994 |  | — | — |
| irAE |  |  |  |  |  |
| No | Reference | — |  | — | — |
| Yes | 1.77 (0.57-5.52) | 0.325 |  | — | — |
| PD-L1(TPS) | 0.99 (0.97-1.01) | 0.480 |  | — | — |
| CEA | 1 (1-1) | 0.736 |  | — | — |
| CYFRA211 | 1 (0.99-1.01) | 0.861 |  | — | — |
| NSE | 1.03 (1-1.05) | 0.026 |  | 1 (0.97-1.03) | 0.838 |
| SCCA | 1.03 (1-1.07) | 0.030 |  | 1.04 (1.01-1.08) | 0.017 |
| WBC | 0.98 (0.86-1.13) | 0.827 |  | — | — |
| MNC | 1.42 (0.32-6.4) | 0.644 |  | — | — |
| LYM | 0.31 (0.1-0.93) | 0.037 |  | 0.32 (0.09-1.18) | 0.086 |
| NEU | 1.02 (0.88-1.18) | 0.805 |  | — | — |
| PLT | 1 (1-1) | 0.751 |  | — | — |
| ALB | 0.91 (0.82-1) | 0.059 |  | — | — |
| CRP | 1 (1-1.01) | 0.003 |  | 1 (1-1.01) | 0.077 |
| LDH | 1 (1-1.01) | 0.307 |  | — | — |

BMI, body mass index; Non, never smoked before; Former, quit smoking for more than 3 months before treatment; Current, still smoking for more than 3 months before treatment; ADC, adenocarcinoma; SCC, squamous cell carcinoma; Other, other pathological types except for adenocarcinoma and squamous cell carcinoma; T, tumor; N, node; M, metastasis; Combination therapy, combination therapy with immune checkpoint inhibitors, i.e., PD1 (e.g., pembrolizumab or nivolumab)/PD-L1 (e.g., durvalumab) ICI in combination with chemotherapeutic agents (e.g., pemetrexed/paclitaxel/gemcitabine+cisplatin/carboplatin/lobaplatin) and/or antiangiogenic agents (e.g., bevacizumab); Monotherapy, immune checkpoint inhibitor monotherapy, i.e., PD1 (e.g., pembrolizumab or nivolumab)/PD-L1 (e.g., durvalumab) ICI monotherapy; irAE, immune-related adverse event; PD-L1(TPS), programmed death-ligand 1(tumor proportion score); CEA, carcinoembryonic antigen; CYFRA211, cytokeratin 19 fragment antigen 211; NSE, neuron-specific enolase; SCCA, squamous cell carcinoma antigen; WBC, white blood cell; MNC, mononuclear cells; LYM, lymphocyte; NEU, neutrophil; PLT, platelet; ALB, albumin; CRP, c-reactive protein; LDH, lactate dehydrogenase; HR, hazard ratio; CI, confidence interval.

**Table S9.** PFS survival analysis of NSCLC patient subgroups stratified by clinical and pathological factors in the training cohort

| **Characteristics** | **COMB-low（%）** | **COMB-high（%）** | **Cox Proportional Hazards Model** | |  | **Log-rank test** |
| --- | --- | --- | --- | --- | --- | --- |
|  |  |  | **HR（95%CI）** | ***p*** |  | ***p*** |
| All parents | 116 | 67 | 4.15 (2.77-6.22) | <0.001 |  | <0.001 |
| Sex |  |  |  |  |  |  |
| Female | 17 (14.66) | 11 (16.42) | 4.04 (1.5-10.89) | 0.006 |  | 0.003 |
| Male | 99 (85.34) | 56 (83.58) | 4.12 (2.65-6.42) | <0.001 |  | <0.001 |
| Age |  |  |  |  |  |  |
| ≤55 | 33 (28.45) | 11 (16.42) | 7.7 (3.15-18.82) | <0.001 |  | <0.001 |
| 56–65 | 42 (36.21) | 27 (40.30) | 3.27 (1.75-6.12) | <0.001 |  | <0.001 |
| >65 | 41 (35.34) | 29 (43.28) | 4.06 (2.07-7.97) | <0.001 |  | <0.001 |
| Smoke |  |  |  |  |  |  |
| Non | 26 (22.41) | 10 (14.93) | 4.71 (1.8-12.34) | 0.002 |  | 0.001 |
| Former | 22 (18.97) | 11 (16.42) | 3.4 (1.33-8.66) | 0.01 |  | 0.007 |
| Current | 68 (58.62) | 46 (68.66) | 4.11 (2.47-6.85) | <0.001 |  | <0.001 |
| Histology |  |  |  |  |  |  |
| ADC | 68 (58.62) | 26 (38.81) | 3.51 (1.97-6.27) | <0.001 |  | <0.001 |
| SCC | 40 (34.48) | 33 (49.25) | 4.64 (2.39-9.01) | <0.001 |  | <0.001 |
| Other | 8 (6.90) | 8 (11.94) | 3.73 (0.93-14.92) | 0.062 |  | 0.053 |
| T stage |  |  |  |  |  |  |
| 1-3 | 63 (54.31) | 29 (43.28) | 4.72 (2.63-8.47) | <0.001 |  | <0.001 |
| 4 | 53 (45.69) | 38 (56.72) | 3.64 (2.08-6.37) | <0.001 |  | <0.001 |
| N stage |  |  |  |  |  |  |
| 0-2 | 40 (34.48) | 20 (29.85) | 3.15 (1.57-6.36) | 0.001 |  | 0.001 |
| 3 | 76 (65.52) | 47 (70.15) | 4.74 (2.88-7.8) | <0.001 |  | <0.001 |
| M stage |  |  |  |  |  |  |
| M0 | 27 (23.28) | 19 (28.36) | 4.08 (1.73-9.63) | 0.001 |  | 0.001 |
| M1a+M1b | 32 (27.59) | 21 (31.34) | 3.14 (1.52-6.5) | 0.002 |  | 0.001 |
| M1c | 57 (49.14) | 27 (40.30) | 5.27 (2.9-9.58) | <0.001 |  | <0.001 |
| Stage |  |  |  |  |  |  |
| III | 27 (23.28) | 19 (28.36) | 4.08 (1.73-9.63) | 0.001 |  | 0.001 |
| IV | 89 (76.72) | 48 (71.64) | 4.18 (2.64-6.62) | <0.001 |  | <0.001 |
| Metastases |  |  |  |  |  |  |
| <3 | 87 (75.00) | 58 (86.57) | 4.17 (2.65-6.55) | <0.001 |  | <0.001 |
| ≥3 | 29 (25.00) | 9 (13.43) | 3.35 (1.29-8.75) | 0.013 |  | 0.009 |
| Treatment strategy |  |  |  |  |  |  |
| Monotherapy | 8 (6.90) | 12 (17.91) | 5 (1.06-23.58) | 0.042 |  | 0.025 |
| Combination therapy | 108 (93.10) | 55 (82.09) | 4.29 (2.79-6.59) | <0.001 |  | <0.001 |
| Local radiotherapy |  |  |  |  |  |  |
| No | 103 (88.79) | 65 (97.01) | 4.09 (2.68-6.24) | <0.001 |  | <0.001 |
| Yes | 13 (11.21) | 2 (2.99) | 15.76 (1.39-178.59) | 0.026 |  | 0.007 |
| irAE |  |  |  |  |  |  |
| No | 95 (81.90) | 60 (89.55) | 4.2 (2.72-6.48) | <0.001 |  | <0.001 |
| Yes | 21 (18.10) | 7 (10.45) | 3.37 (1.06-10.7) | 0.039 |  | 0.029 |

Former, quit smoking for more than 3 months before treatment; Current, still smoking for more than 3 months before treatment; ADC, adenocarcinoma; SCC, squamous cell carcinoma; Other, other pathological types except for adenocarcinoma and squamous cell carcinoma; T, tumor; N, node; M, metastasis; Combination therapy, combination therapy with immune checkpoint inhibitors, i.e., PD1 (e.g., pembrolizumab or nivolumab)/PD-L1 (e.g., durvalumab) ICI in combination with chemotherapeutic agents (e.g., pemetrexed/paclitaxel/gemcitabine+cisplatin/carboplatin/lobaplatin) and/or antiangiogenic agents (e.g., bevacizumab); Monotherapy, immune checkpoint inhibitor monotherapy, i.e., PD1 (e.g., pembrolizumab or nivolumab)/PD-L1 (e.g., durvalumab) ICI monotherapy; irAE, immune-related adverse event.

**Table S10.** PFS survival analysis of NSCLC patient subgroups stratified by clinical and pathological factors in the testing cohort

| **Characteristics** | **COMB-low（%）** | **COMB-high（%）** | **Cox Proportional Hazards Model** | |  | **Log-rank test** |
| --- | --- | --- | --- | --- | --- | --- |
|  |  |  | **HR（95%CI）** | ***p*** |  | ***p*** |
| All parents | 53 | 25 | 2.95 (1.54-5.63) | 0.001 |  | <0.001 |
| Sex |  |  |  |  |  |  |
| Female | 8 (15.09) | 8 (32.00) | 3.98 (0.96-16.47) | 0.057 |  | 0.043 |
| Male | 45 (84.91) | 17 (68.00) | 2.8 (1.29-6.09) | 0.009 |  | 0.007 |
| Age |  |  |  |  |  |  |
| ≤55 | 11 (20.75) | 5 (20.00) | 12.6 (2.34-67.97) | 0.003 |  | <0.001 |
| 56–65 | 17 (32.08) | 9 (36.00) | 4.71 (1.21-18.28) | 0.025 |  | 0.015 |
| >65 | 25 (47.17) | 11 (44.00) | 2.37 (0.88-6.43) | 0.089 |  | 0.080 |
| Smoke |  |  |  |  |  |  |
| Non | 13 (24.53) | 10 (40.00) | 3.26 (1.02-10.44) | 0.046 |  | 0.036 |
| Former | 12 (22.64) | 3 (12.00) | 1.36 (0.14-13.11) | 0.793 |  | 0.792 |
| Current | 28 (52.83) | 12 (48.00) | 3.03 (1.27-7.22) | 0.012 |  | 0.009 |
| Histology |  |  |  |  |  |  |
| ADC | 34 (64.15) | 18 (72.00) | 2.95 (1.35-6.48) | 0.007 |  | 0.005 |
| SCC | 12 (22.64) | 5 (20.00) | 2.83 (0.63-12.71) | 0.174 |  | 0.156 |
| Other | 7 (13.21) | 2 (8.00) | 4.58 (0.28-75.13) | 0.286 |  | 0.243 |
| T stage |  |  |  |  |  |  |
| 1-3 | 35 (66.04) | 10 (40.00) | 2.1 (0.79-5.56) | 0.136 |  | 0.128 |
| 4 | 18 (33.96) | 15 (60.00) | 2.72 (1.08-6.84) | 0.033 |  | 0.027 |
| N stage |  |  |  |  |  |  |
| 0-2 | 21 (39.62) | 6 (24.00) | 3.81 (1.04-13.95) | 0.043 |  | 0.030 |
| 3 | 32 (60.38) | 19 (76.00) | 2.98 (1.35-6.59) | 0.007 |  | 0.005 |
| M stage |  |  |  |  |  |  |
| M0 | 17 (32.08) | 8 (32.00) | 18.88 (3.91-91.25) | <0.001 |  | <0.001 |
| M1a+M1b | 20 (37.74) | 6 (24.00) | 3.24 (1.07-9.88) | 0.038 |  | 0.031 |
| M1c | 16 (30.19) | 11 (44.00) | 0.96 (0.29-3.21) | 0.952 |  | 0.952 |
| Stage |  |  |  |  |  |  |
| III | 17 (32.08) | 8 (32.00) | 18.88 (3.91-91.25) | <0.001 |  | <0.001 |
| IV | 36 (67.92) | 17 (68.00) | 1.59 (0.71-3.55) | 0.261 |  | 0.265 |
| Metastases |  |  |  |  |  |  |
| <3 | 43 (81.13) | 17 (68.00) | 2.83 (1.38-5.81) | 0.005 |  | 0.003 |
| ≥3 | 10 (18.87) | 8 (32.00) | 2.64 (0.57-12.2) | 0.214 |  | 0.198 |
| Treatment strategy |  |  |  |  |  |  |
| Monotherapy | 4 (7.55) | 4 (16.00) | 0.31 (0.03-2.98) | 0.31 |  | 0.283 |
| Combination therapy | 49 (92.45) | 21 (84.00) | 4.81 (2.4-9.64) | <0.001 |  | <0.001 |
| Local radiotherapy |  |  |  |  |  |  |
| No | 43 (81.13) | 25 (100.00) | 2.97 (1.49-5.92) | 0.002 |  | 0.001 |
| Yes | 10 (18.87) | 0 (0.00) | NA | NA |  | NA |
| irAE |  |  |  |  |  |  |
| No | 45 (84.91) | 22 (88.00) | 2.87 (1.41-5.83) | 0.004 |  | 0.003 |
| Yes | 8 (15.09) | 3 (12.00) | 2.35 (0.46-12.02) | 0.303 |  | 0.290 |

Former, quit smoking for more than 3 months before treatment; Current, still smoking for more than 3 months before treatment; ADC, adenocarcinoma; SCC, squamous cell carcinoma; Other, other pathological types except for adenocarcinoma and squamous cell carcinoma; T, tumor; N, node; M, metastasis; Combination therapy, combination therapy with immune checkpoint inhibitors, i.e., PD1 (e.g., pembrolizumab or nivolumab)/PD-L1 (e.g., durvalumab) ICI in combination with chemotherapeutic agents (e.g., pemetrexed/paclitaxel/gemcitabine+cisplatin/carboplatin/lobaplatin) and/or antiangiogenic agents (e.g., bevacizumab); Monotherapy, immune checkpoint inhibitor monotherapy, i.e., PD1 (e.g., pembrolizumab or nivolumab)/PD-L1 (e.g., durvalumab) ICI monotherapy; irAE, immune-related adverse event.

**Table S11.** Positive Predictive Value (PPV), Negative Predictive Value (NPV), Sensitivity, Specificity, Accuracy, Recall, F1 Score, Matthews Correlation Coefficient (MCC), and Kappa for COMB-Radscore (Follow-up) in the follow-up cohorts

|  | **Follow-up cohort (N=25)** |
| --- | --- |
|  | **COMB-Radscore (Follow-up)** |
| Cut-off Value | -0.617 |
| TP | 9 |
| TN | 11 |
| FP | 3 |
| FN | 2 |
| PPV | 0.75 |
| NPV | 0.85 |
| Sensitivity | 0.82 |
| Specificity | 0.79 |
| Accuracy | 0.80 |
| Precision | 0.75 |
| Recall | 0.82 |
| F1 score | 0.78 |
| MCC | 0.60 |
| Kappa | 0.60 |

TP, true positives; TN, true negatives; FP, false positives; FN, false negatives; PPV, positive predictive value; NPV, negative predictive value; MCC, matthews correlation coefficient.

**Table S12.** Consistency and correlation of radiomic features between primary lung cancer and liver metastases

| **Radiomic features** | **ICC** | |  | **Correlation** | |
| --- | --- | --- | --- | --- | --- |
|  | **ICC Value** | ***p*** |  | **R Value** | ***p*** |
| PET/CT Intratumor wavelet LLH firstorder Mean | 0.068 | 0.361 |  | 0.015 | 0.938 |
| PET TumorPeritumor log sigma 4.0 mm 3D glszm GrayLevelNonUniformityNormalized | 0.269 | 0.077 |  | 0.258 | 0.184 |
| CT Intratumor lbp 3D k firstorder Variance | 0.174 | 0.181 |  | 0.276 | 0.155 |
| PET Tumor wavelet LLH gldm HighGrayLevelEmphasis | -0.399 | 0.984 |  | -0.436 | 0.020 |
| PET/CT Tumor wavelet HHL glcm MCC | 0.260 | 0.084 |  | 0.176 | 0.370 |
| CT Intratumor log sigma 1.0 mm 3D gldm DependenceVariance | 0.453 | 0.006 |  | 0.396 | 0.037 |
| PET/CT TumorPeritumor wavelet HHH glszm HighGrayLevelZoneEmphasis | 0.367 | 0.024 |  | 0.408 | 0.031 |
| PET/CT Intratumor original glszm GrayLevelNonUniformity | -0.023 | 0.546 |  | 0.071 | 0.721 |
| PET Intratumor wavelet HHH firstorder InterquartileRange | -0.021 | 0.543 |  | 0.353 | 0.065 |
| PET/CT TumorPeritumor wavelet LLL gldm DependenceVariance | 0.033 | 0.432 |  | 0.057 | 0.771 |
| PET/CT Intratumor wavelet HLH firstorder 10Percentile | 0.455 | 0.006 |  | 0.506 | 0.006 |
| PET Intratumor wavelet LLH glcm ClusterShade | -0.071 | 0.642 |  | -0.047 | 0.814 |
| PET Tumor log sigma 5.0 mm 3D glszm LowGrayLevelZoneEmphasis | 0.156 | 0.207 |  | 0.202 | 0.303 |
| PET/CT Intratumor square glszm SizeZoneNonUniformityNormalized | 0.056 | 0.385 |  | 0.049 | 0.804 |
| PET TumorPeritumor wavelet HLH firstorder Skewness | 0.003 | 0.492 |  | 0.085 | 0.666 |
| PET Tumor lbp 3D m1 glrlm ShortRunLowGrayLevelEmphasis | 0.165 | 0.194 |  | 0.087 | 0.660 |
| PET Peritumor wavelet LLH glcm ClusterShade | -0.053 | 0.608 |  | -0.240 | 0.218 |
| PET Tumor wavelet LHH glcm ClusterShade | 0.126 | 0.255 |  | 0.088 | 0.658 |
| PET Tumor wavelet HHL gldm LargeDependenceLowGrayLevelEmphasis | 0.258 | 0.086 |  | 0.068 | 0.731 |
| PET/CT Peritumor exponential ngtdm Strength | 0.010 | 0.479 |  | -0.328 | 0.088 |
| PET/CT Intratumor original gldm SmallDependenceHighGrayLevelEmphasis | 0.302 | 0.053 |  | 0.371 | 0.052 |
| PET TumorPeritumor log sigma 5.0 mm 3D glszm GrayLevelNonUniformityNormalized | 0.294 | 0.059 |  | 0.056 | 0.776 |
| PET TumorPeritumor log sigma 1.0 mm 3D glszm SmallAreaLowGrayLevelEmphasis | -0.232 | 0.888 |  | -0.189 | 0.334 |
| PET/CT Peritumor wavelet HLH glrlm LongRunHighGrayLevelEmphasis | 0.049 | 0.398 |  | -0.025 | 0.901 |
| PET Peritumor log sigma 5.0 mm 3D glrlm ShortRunEmphasis | 0.528 | 0.001 |  | 0.525 | 0.004 |

ICC, intraclass correlation coefficient.

**Table S13.** Consistency and correlation of radiomic features between primary lung cancer and adrenal metastases

| **Radiomic features** | **ICC** | |  | **Correlation** | |
| --- | --- | --- | --- | --- | --- |
|  | **ICC Value** | ***p*** |  | **R Value** | ***p*** |
| PET/CT Intratumor wavelet LLH firstorder Mean | 0.104 | 0.270 |  | 0.099 | 0.573 |
| PET TumorPeritumor log sigma 4.0 mm 3D glszm GrayLevelNonUniformityNormalized | -0.112 | 0.742 |  | -0.112 | 0.523 |
| CT Intratumor lbp 3D k firstorder Variance | 0.351 | 0.017 |  | 0.398 | 0.018 |
| PET Tumor wavelet LLH gldm HighGrayLevelEmphasis | 0.008 | 0.481 |  | -0.055 | 0.754 |
| PET/CT Tumor wavelet HHL glcm MCC | 0.076 | 0.327 |  | -0.018 | 0.919 |
| CT Intratumor log sigma 1.0 mm 3D gldm DependenceVariance | -0.252 | 0.932 |  | -0.197 | 0.256 |
| PET/CT TumorPeritumor wavelet HHH glszm HighGrayLevelZoneEmphasis | -0.164 | 0.832 |  | -0.331 | 0.052 |
| PET/CT Intratumor original glszm GrayLevelNonUniformity | 0.040 | 0.407 |  | -0.107 | 0.540 |
| PET Intratumor wavelet HHH firstorder InterquartileRange | -0.029 | 0.566 |  | 0.011 | 0.950 |
| PET/CT TumorPeritumor wavelet LLL gldm DependenceVariance | -0.020 | 0.546 |  | -0.019 | 0.915 |
| PET/CT Intratumor wavelet HLH firstorder 10Percentile | 0.030 | 0.430 |  | 0.164 | 0.345 |
| PET Intratumor wavelet LLH glcm ClusterShade | 0.249 | 0.069 |  | 0.281 | 0.102 |
| PET Tumor log sigma 5.0 mm 3D glszm LowGrayLevelZoneEmphasis | 0.295 | 0.039 |  | 0.313 | 0.067 |
| PET/CT Intratumor square glszm SizeZoneNonUniformityNormalized | -0.072 | 0.662 |  | -0.103 | 0.555 |
| PET TumorPeritumor wavelet HLH firstorder Skewness | 0.050 | 0.385 |  | 0.160 | 0.360 |
| PET Tumor lbp 3D m1 glrlm ShortRunLowGrayLevelEmphasis | 0.150 | 0.188 |  | 0.196 | 0.258 |
| PET Peritumor wavelet LLH glcm ClusterShade | 0.109 | 0.261 |  | 0.208 | 0.231 |
| PET Tumor wavelet LHH glcm ClusterShade | -0.408 | 0.994 |  | -0.136 | 0.437 |
| PET Tumor wavelet HHL gldm LargeDependenceLowGrayLevelEmphasis | 0.018 | 0.459 |  | 0.107 | 0.542 |
| PET/CT Peritumor exponential ngtdm Strength | -0.004 | 0.508 |  | -0.132 | 0.448 |
| PET/CT Intratumor original gldm SmallDependenceHighGrayLevelEmphasis | 0.209 | 0.109 |  | 0.204 | 0.239 |
| PET TumorPeritumor log sigma 5.0 mm 3D glszm GrayLevelNonUniformityNormalized | -0.285 | 0.955 |  | -0.248 | 0.151 |
| PET TumorPeritumor log sigma 1.0 mm 3D glszm SmallAreaLowGrayLevelEmphasis | 0.040 | 0.408 |  | 0.011 | 0.949 |
| PET/CT Peritumor wavelet HLH glrlm LongRunHighGrayLevelEmphasis | 0.158 | 0.176 |  | 0.058 | 0.742 |
| PET Peritumor log sigma 5.0 mm 3D glrlm ShortRunEmphasis | 0.098 | 0.284 |  | 0.089 | 0.611 |

ICC, intraclass correlation coefficient.

|  | **Sub-training cohort（N=128）** | | | |  | **Sub-testing cohort（N=54）** | | | |
| --- | --- | --- | --- | --- | --- | --- | --- | --- | --- |
|  | **NRI (95%CI)** | ***p*** | **IDI (95% CI)** | ***p*** |  | **NRI (95% CI)** | ***p*** | **IDI (95% CI)** | ***p*** |
| TPS-Radscore vs COMB-Radscore (Reference) | 0.22  （0.07，0.36） | 0.002 | 0.35  （0.28，0.42） | <0.001 |  | 0.10  （-0.10，0.30） | 0.330 | 0.25  （0.13，0.36） | <0.001 |

**Table S14.** NRI and IDI analysis of TPS-Radscore and COMB-Radscore

CI, confidence interval; NRI, net reclassification improvement; IDI, integrated discrimination improvement.

**Table S15.** Positive Predictive Value (PPV), Negative Predictive Value (NPV), Sensitivity, Specificity, Accuracy, Recall, F1 Score, Matthews Correlation Coefficient (MCC), and Kappa for COMB-Radscore and TPS- Radscore

|  | **Sub-training cohort（N=128）** | |  | **Sub-testing cohort（N=54）** | |
| --- | --- | --- | --- | --- | --- |
|  | **COMB-Radscore** | **TPS-Radscore** |  | **COMB-Radscore** | **TPS-Radscore** |
| Cut-off Value | -0.617 | 0.167 |  | -0.617 | 0.167 |
| TP | 35 | 41 |  | 10 | 12 |
| TN | 68 | 75 |  | 31 | 30 |
| FP | 16 | 9 |  | 7 | 8 |
| FN | 9 | 3 |  | 6 | 4 |
| PPV | 0.686 | 0.820 |  | 0.588 | 0.6 |
| NPV | 0.883 | 0.962 |  | 0.838 | 0.882 |
| Sensitivity | 0.795 | 0.932 |  | 0.625 | 0.75 |
| Specificity | 0.810 | 0.893 |  | 0.816 | 0.789 |
| Accuracy | 0.805 | 0.906 |  | 0.759 | 0.778 |
| Precision | 0.686 | 0.820 |  | 0.588 | 0.6 |
| Recall | 0.795 | 0.932 |  | 0.625 | 0.75 |
| F1score | 0.737 | 0.872 |  | 0.606 | 0.667 |
| MCC | 0.587 | 0.803 |  | 0.433 | 0.51 |
| Kappa | 0.583 | 0.799 |  | 0.433 | 0.503 |

TP, true positives; TN, true negatives; FP, false positives; FN, false negatives; PPV, positive predictive value; NPV, negative predictive value; MCC, matthews correlation coefficient.

**Table S16.** The detailed description of PET, CT and PET/CT image preprocessing and generation of derived images for customizing extraction in Pyradiomics

| **^18^F-FDG PET/CT image preprocessing** | | | |
| --- | --- | --- | --- |
| Parameter | PET | CT | PET/CT |
| Interpolation |  |  |  |
| Interpolation method | B-spline | B-spline | B-spline |
| Resampled voxel spacing | 1×1×1 mm | 1×1×1 mm | 1×1×1 mm |
| Discretization |  |  |  |
| Discretization method | FBC | FBS | FBS |
| Bin size | 32（bin count） | 25（bin width） | 25（bin width） |
| geometryTolerance | 0.0001 | 0.0001 | 0.0001 |
| removeOutliers | 3 | 3 | 3 |
| **Generation of derived images** | | | |
| **Image Type** |  | | |
| Original | No filter applied. | | |
| Square | Takes the square of the image intensities and linearly scales them back to the original range. Negative values in the original image will be made negative again after application of filter. | | |
| Square Root | Takes the square root of the absolute image intensities and scales them back to original range. Negative values in the original image will be made negative again after application of filter. | | |
| Logarithm | Takes the logarithm of the absolute intensity + 1. Values are scaled to original range and negative original values are made negative again after application of filter. | | |
| Exponential | Takes the exponential, where filtered intensity is e^(absolute intensity). Values are scaled to original range and negative original values are made negative again after application of filter. | | |
| Gradient | Returns the magnitude of the local gradient. | | |
| Local Binary Pattern 3D（LBP） | Computes the Local Binary Pattern in 3D using spherical harmonics. | | |
| Laplacian of Gaussian  (LoG) | Laplacian of Gaussian filter, edge enhancement filter. Emphasizes areas of gray level change, where sigma defines how coarse the emphasised texture should be. A low sigma emphasis on fine textures (change over a short distance), where a high sigma value emphasises coarse textures (gray level change over a large distance). The sigma value is set to 1 mm, 2 mm, 3 mm, 4 mm and 5 mm. | | |
| Wavelet | Wavelet filtering, yields 8 decompositions per level (all possible combinations of applying either a High or a Low pass filter in each of the three dimensions. The wavelet basis function is rbio1.1. | | |

^18^F-FDG PET/CT, fluoro-18-fluorodeoxyglucose positron emission tomography/computed tomography; FBC, fixed bins count; FBS, fixed bins size; LBP, local binary pattern, LoG, laplacian of gaussian.

**Table S17.** List of extracted radiomic features

| **Feature Class (Extraction description)** | | **Feature name** |
| --- | --- | --- |
| **Shape**  **(14 features)** | 1 | Elongation |
|  | 2 | Flatness |
|  | 3 | Least Axis Length |
|  | 4 | Major Axis Length |
|  | 5 | Maximum 2D Diameter (Column) |
|  | 6 | Maximum 2D Diameter (Row) |
|  | 7 | Maximum 2D Diameter (Slice) |
|  | 8 | Maximum 3D Diameter |
|  | 9 | Mesh Volume |
|  | 10 | Minor Axis Length |
|  | 11 | Sphericity |
|  | 12 | Surface Area |
|  | 13 | Surface Area to Volume Ratio |
|  | 14 | Voxel Volume |
| **First-order**  **(18 features)** | 1 | 10th percentile |
|  | 2 | 90th percentile |
|  | 3 | Energy |
|  | 4 | Entropy |
|  | 5 | Interquartile Range |
|  | 6 | Kurtosis |
|  | 7 | Maximum |
|  | 8 | Mean Absolute Deviation |
|  | 9 | Mean |
|  | 10 | Median |
|  | 11 | Minimum |
|  | 12 | Range |
|  | 13 | Robust Mean Absolute Deviation |
|  | 14 | Root Mean Squared |
|  | 15 | Skewness |
|  | 16 | Total Energy |
|  | 17 | Uniformity |
|  | 18 | Variance |
| **Texture - Gray Level Cooccurrence Matrix (GLCM) Features**  **(24 features)** | 1 | Autocorrelation |
|  | 2 | Cluster Prominence |
|  | 3 | Cluster Shade |
|  | 4 | Cluster Tendency |
|  | 5 | Contrast |
|  | 6 | Correlation |
|  | 7 | Difference Average |
|  | 8 | Difference Entropy |
|  | 9 | Difference Variance |
|  | 10 | Inverse Difference |
|  | 11 | Inverse Difference Moment |
|  | 12 | Inverse Difference Moment Normalized |
|  | 13 | Inverse Difference Normalized |
|  | 14 | Informational Measure of Correlation 1 |
|  | 15 | Informational Measure of Correlation 2 |
|  | 16 | Inverse Variance |
|  | 17 | Joint Average |
|  | 18 | Joint Energy |
|  | 19 | Joint Entropy |
|  | 20 | Maximal Correlation Coefficient |
|  | 21 | Maximum Probability |
|  | 22 | Sum Average |
|  | 23 | Sum Entropy |
|  | 24 | Sum of Squares |
| **Texture - Gray Level Size Zone Matrix (GLSZM) Features**  **(16 features)** | 1 | Gray Level Non-Uniformity |
|  | 2 | Gray Level Non-Uniformity Normalized |
|  | 3 | Gray Level Variance |
|  | 4 | High Gray Level Zone Emphasis |
|  | 5 | Large Area Emphasis |
|  | 6 | Large Area High Gray Level Emphasis |
|  | 7 | Large Area Low Gray Level Emphasis |
|  | 8 | Low Gray Level Zone Emphasis |
|  | 9 | Size Zone Non-Uniformity |
|  | 10 | Size Zone Non-Uniformity Normalized |
|  | 11 | Small Area Emphasis |
|  | 12 | Small Area High Gray Level Emphasis |
|  | 13 | Small Area Low Gray Level Emphasis |
|  | 14 | Zone Entropy |
|  | 15 | Zone Percentage |
|  | 16 | Zone Variance |
| **Texture - Gray Level Run Length Matrix (GLRLM) Features**  **(16 features)** | 1 | Gray Level Non-Uniformity |
|  | 2 | Gray Level Non-Uniformity Normalized |
|  | 3 | Gray Level Variance |
|  | 4 | High Gray Level Run Emphasis |
|  | 5 | Long Run Emphasis |
|  | 6 | Long Run High Gray Level Emphasis |
|  | 7 | Long Run Low Gray Level Emphasis |
|  | 8 | Low Gray Level Run Emphasis |
|  | 9 | Run Entropy |
|  | 10 | Run Length Non-Uniformity |
|  | 11 | Run Length Non-Uniformity Normalized |
|  | 12 | Run Percentage |
|  | 13 | Run Variance |
|  | 14 | Short Run Emphasis |
|  | 15 | Short Run High Gray Level Emphasis |
|  | 16 | Short Run Low Gray Level Emphasis |
| **Texture - Neighboring Gray Tone Difference Matrix (NGTDM) Features**  **(5 features)** | 1 | Busyness |
|  | 2 | Coarseness |
|  | 3 | Complexity |
|  | 4 | Contrast |
|  | 5 | Strength |
| **Texture - Gray Level Dependence Matrix (GLDM) Features**  **(14 features)** | 1 | Dependence Entropy |
|  | 2 | Dependence Non-Uniformity |
|  | 3 | Dependence Non-Uniformity Normalized |
|  | 4 | Dependence Variance |
|  | 5 | Gray Level Non-Uniformity |
|  | 6 | Gray Level Variance |
|  | 7 | High Gray Level Emphasis |
|  | 8 | Large Dependence Emphasis |
|  | 9 | Large Dependence High Gray Level Emphasis |
|  | 10 | Large Dependence Low Gray Level Emphasis |
|  | 11 | Low Gray Level Emphasis |
|  | 12 | Small Dependence Emphasis |
|  | 13 | Small Dependence High Gray Level Emphasis |
|  | 14 | Small Dependence Low Gray Level Emphasis |

GLCM, gray-level co-occurrence matrix; GLDM, gray-level dependence matrix; GLRLM, gray-level run-length matrix; GLSZM, gray-level size-zone matrix; NGTDM, neighboring gray-tone difference matrix.

Exact feature definitions are provided in <https://pyradiomics.readthedocs.io/>.
