# Supplementary material for: Intratumoral and peritumoral PET/CT-based radiomics for non-invasively and dynamically predicting immunotherapy response in NSCLC
Source: Br J Cancer. 2025 Feb 10;132(6):558–68. doi: 10.1038/s41416-025-02948-z (PMC11920075; doi:10.1038/s41416-025-02948-z)

Figure legends

Original ROC Curves

Figure 2A ROC curves of the four radiomic models in the testing cohort

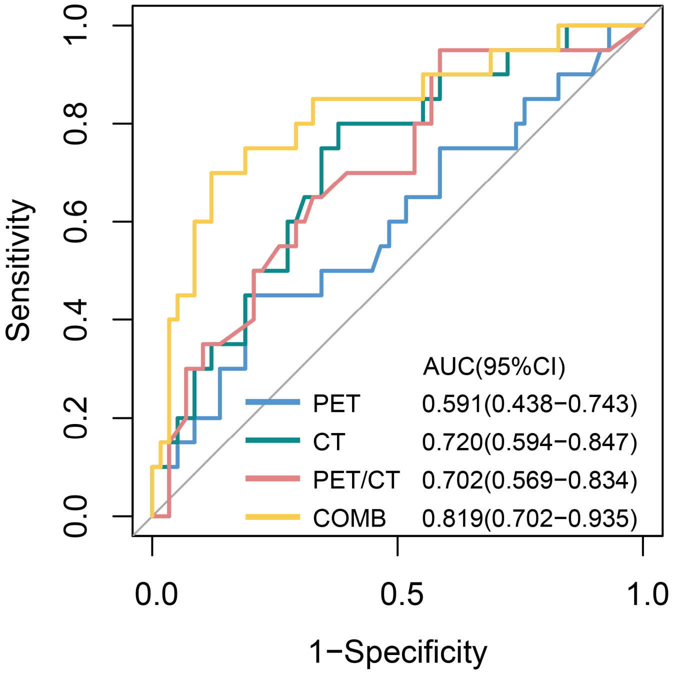

Figure 4A ROC curve of COMB-Radscore (Follow-up) in the follow-up cohort

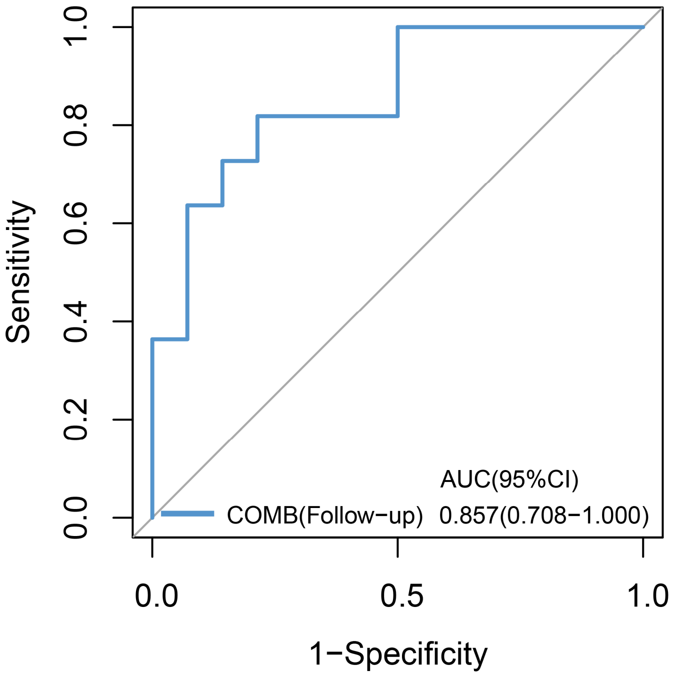

Figure 5A ROC curve of TPS-Lung in the COMB-Radscore prediction failure cohort

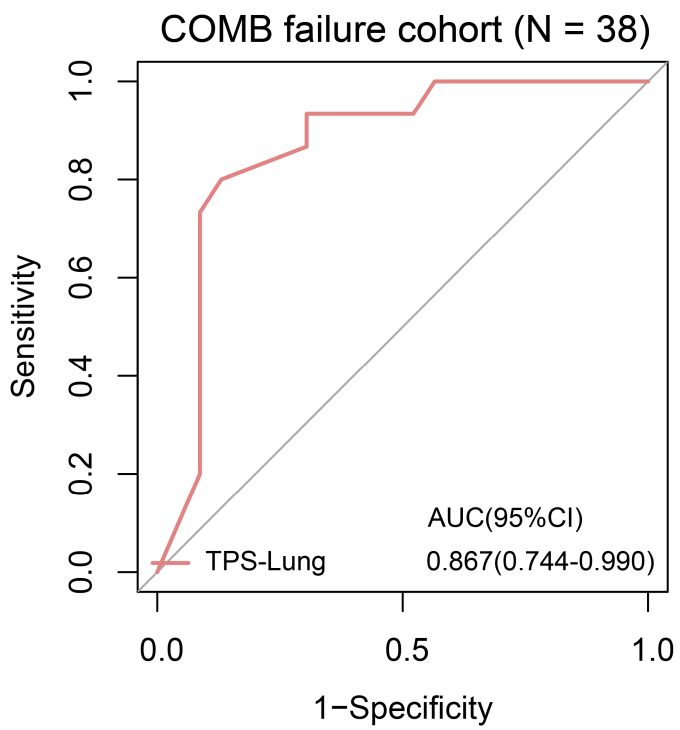

Figure 5B ROC curve of COMB-Radscore in the TPS-Lung prediction failure cohort

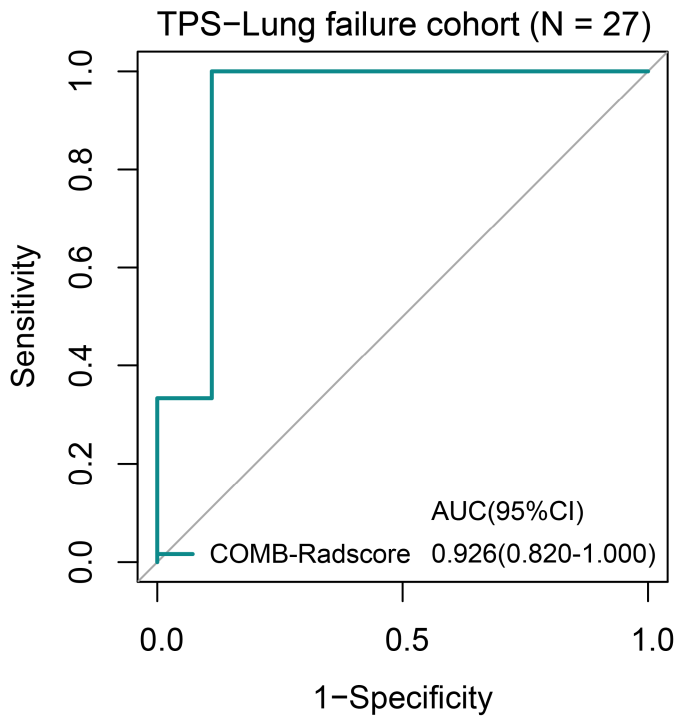

Figure 5D ROC curves of the TPS-Radscore, COMB-Radscore, and TPS-Lung in the sub-training (left) and sub-testing (right) cohorts

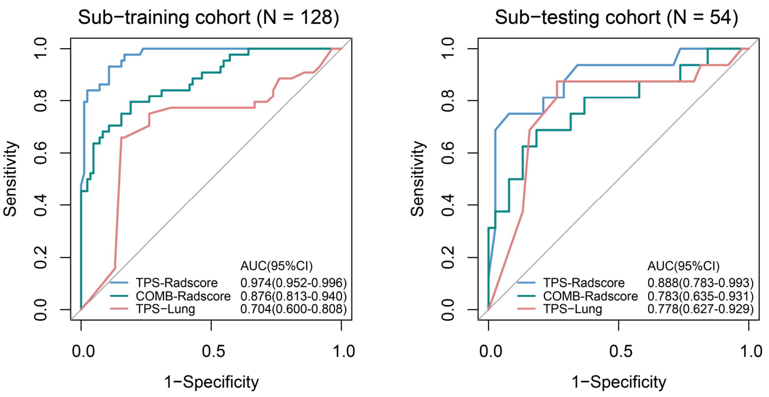

Figure S2A ROC curves of the four radiomic models in the training cohort

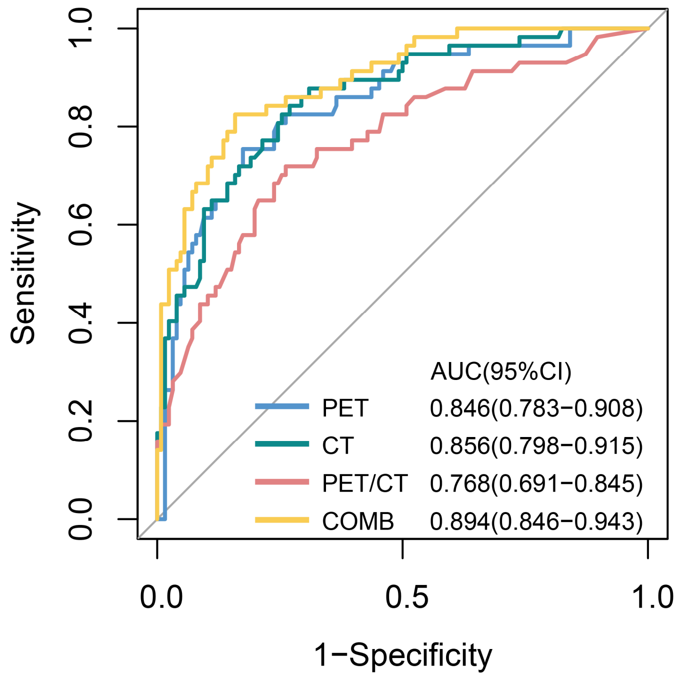

Figure S4A ROC curves of 10 serum inflammatory markers and COMB-Radscore in the training cohort

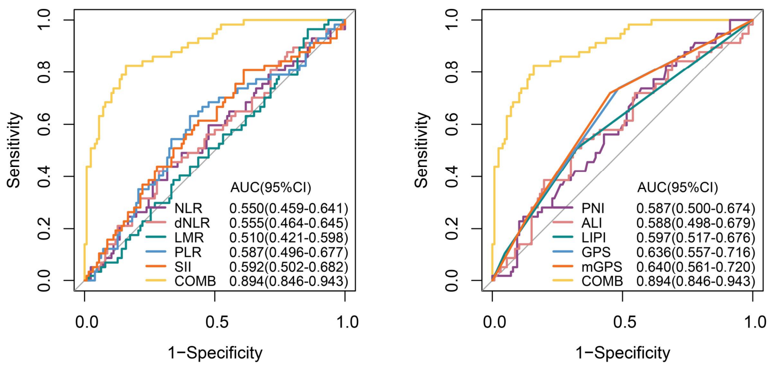

Figure S4B ROC curves of 10 serum inflammatory markers and COMB-Radscore in the testing cohort

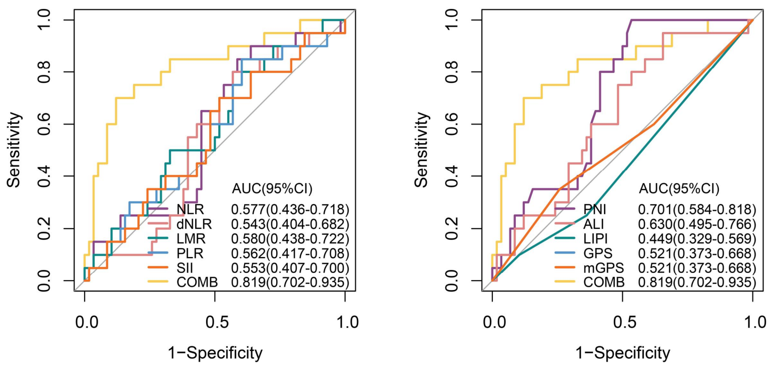

Figure S6A ROC curves of PDL1(TPS) based on different biopsy sites (primary lung tumor, LN metastases and other organ metastases)

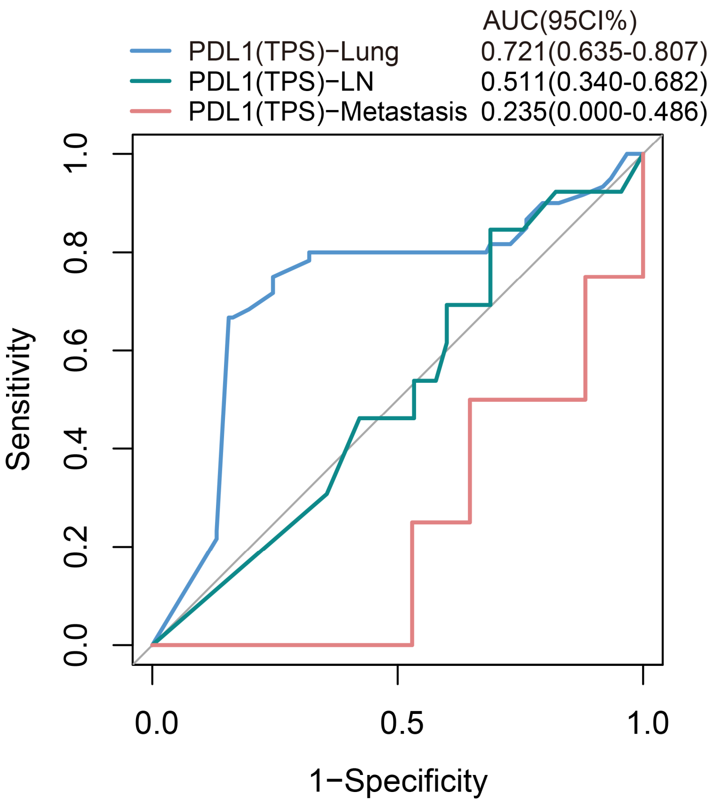

Figure S7A ROC curves of COMB-Radscore based on liver metastases and primary lung tumor

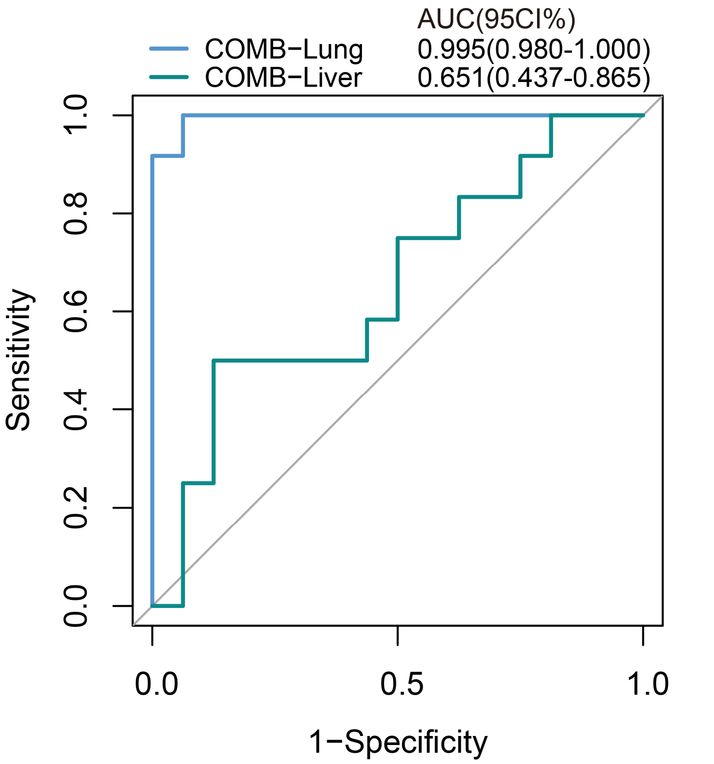

Figure S8A ROC curves of COMB-Radscore based on adrenal metastases and primary lung tumor

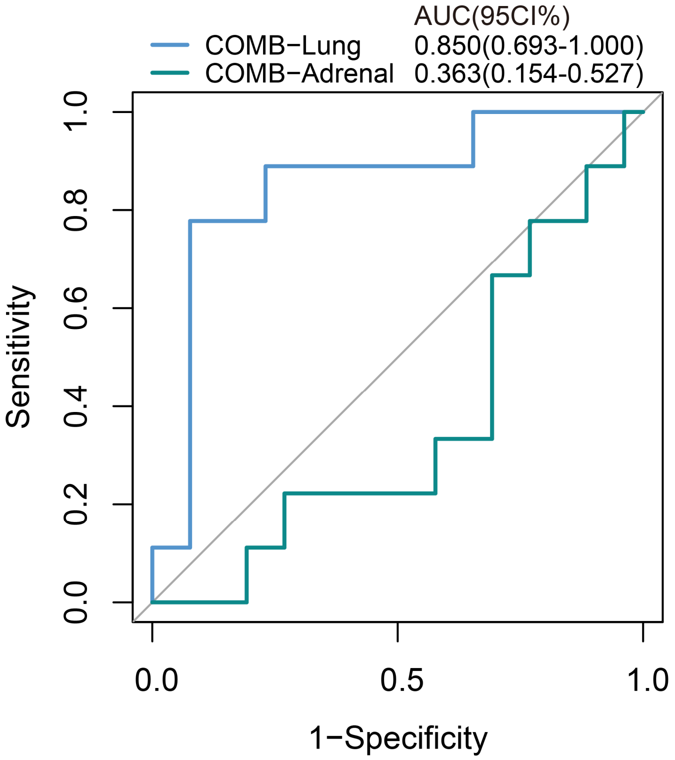

Supplement: Supplementary file 5 — Supplementary Material(Original ROC Curves) [file 41416_2025_2948_MOESM5_ESM.pdf]
